# The Pd (II) Reduction Mechanisms in *Bacillus megaterium* Y-4 Revealed by Proteomic Analysis

**DOI:** 10.3390/nano14060512

**Published:** 2024-03-12

**Authors:** Yuan Chen, Jiaxing Wang, Daidi Chen, Boxi Wang, Jinchuan Wu, Rongrong Liu, Qingxin Li

**Affiliations:** 1 Guangdong Provincial Engineering Laboratory of Biomass High Value Utilization, Institute of Biological and Medical Engineering, Guangdong Academy of Sciences, Guangzhou 510316, China; 2 Key Laboratory for Water Quality and Conservation of the Pearl River Delta, Institute of Environmental Research at Greater Bay Area, Ministry of Education, Guangzhou University, Guangzhou 510006, China; 3 Ministry of Education Key Laboratory of Pollution Control and Ecological Remediation for Industrial Agglomeration Area, School of Environment and Energy, South China University of Technology, Guangzhou 510006, China; 19120332369@163.com

**Keywords:** palladium, biological reduction, protein expression, proteomics, *Bacillus megaterium* Y-4

## Abstract

Many studies have been conducted on the microbial reduction of Pd (II) to palladium nanoparticles (Pd-NPs) due to the environmental friendliness, low cost, and the decreased toxicity of Pd (II) ions. In this study, we investigate the reduction mechanism of Pd (II) by *Bacillus megaterium* Y-4 through proteomics. The data are available via ProteomeXchange with identifier PXD049711. Our results revealed that *B. megaterium* Y-4 may use the endogenous electron donor (NAD(P)H) generated by nirB, tdh, and fabG and reductase to reduce Pd (II) to Pd-NPs. The expression levels of fabG, tdh, gudB, and rocG that generate NAD(P)H were further increased, and the number of reduced Pd-NPs was further increased with the exogenous electron donor sodium formate. Endogenous electron mediators such as quinones and flavins in *B. megaterium* Y-4 can further enhance Pd (II) reduction. The findings provided invaluable information regarding the reduction mechanism of Pd (II) by *B. megaterium* Y-4 at the proteome level.

## 1. Introduction

Palladium (Pd) is a valuable metal and has been widely applied in pharmaceutics, electroplating, and precision manufacturing due to its excellent catalytic performance, excellent effectiveness, and good stability [1]. However, palladium is extremely rare and expensive [2]. At the same time, the discharge of palladium-containing waste impacts the environmental ecosystem and threatens human health [3]. Therefore, researchers have been motivated to develop diverse methods to effectively recover and recycle Pd from different sources [4]. Some microorganisms (e.g., *Geobacter sulfurreducens* [4,5], *Shewanella oneidensis* [6,7], *Enterococcus faecalis* [8], and *Enterobacter cloacae* [9]) can convert Pd (II) through a reduction reaction under room temperature and pressure [10]. Compared to conventional physical and chemical methods, the microbial reduction of Pd (II) is renewable and low-cost without introducing contamination [11]. Pd (0) nanoparticles (Pd-NPs) can be bio-synthesized in periplasmic, intracellular, or extracellular space [12]. Biosynthesized palladium nanoparticles can play a role in the reduction and removal of Cr (VI) [13], the dehalogenation of polychlorinated dioxins [14], the Mizoroki–Heck reaction, etc. [15].

Pd (II) reduction can be achieved through several pathways. In the reduction reaction, Pd (II) serves as a respiratory terminal electron acceptor when dissimilatory metal-reducing bacteria (DMRB) oxidize organic matter and then transfer the released electrons to Pd (II) for respiration, resulting in the bio-reduction to Pd-NPs [9,16]. The role of the NADH dehydrogenases and hydrogenases (especially HyaB) of *Shewanella oneidensis* MR-1 was demonstrated to be responsible for the formation of bio-Pd on the outer membrane and in the periplasmic space, respectively [17]. Transcriptomic analyses revealed that the genes encoding NADH–quinone oxidoreductase, dehydrogenases, cytochrome c reductase, cytochrome c oxidase, the quinone cycle, and ribE in *Bacillus thuringiensis* Y9 have a strong positive relationship with palladium reduction [18]. The genes encoding oxidoreductase, metabolic enzymes, and cell-surface proteins were mainly identified to be responsible for the reduction of Pd (II) [19]. Further investigation of the microbial reduction mechanism of Pd (II) and the identification of related proteins will provide insights into the microbial reduction of palladium.

Our previous research demonstrated that the aerobic bacteria *B. megaterium* Y-4 can reduce Pd (II) [12], but it is unclear which enzymes and proteins are involved in this process. Proteomics is a powerful tool to monitor protein levels and has been applied to elucidate the cellular molecular mechanism of organisms responding to environmental stress induced by different conditions. Analyzing differentially expressed proteins (DEPs) is a strategy used to identify proteins involved in the response [20]. Here, to elucidate the Pd (II) reduction mechanisms in *B. megaterium* Y-4, we analyzed the proteomes of *B. megaterium* Y-4 after Pd (II) exposure under anaerobic conditions in the absence (N_2_) and presence of 5 mM sodium formate (N_2_-SF). The DEPs were identified by high-resolution liquid chromatography–tandem mass spectrometry (LC-MS/MS). Our results showed that the nirB, tdh, and fabG that are responsible for NAD(P)H production were upregulated under both conditions. *B. megaterium* Y-4 can use NAD(P)H to provide electrons for Pd (II) reduction. The higher expression level of gudB and zwf proteins that produce NAD(P)H in N_2_-SF than in N_2_ may partially explain the higher Pd (II) reduction in N_2_-SF than N_2_. Pd (II) may also be reduced by the ssuE, trx-1, and flavin reductase family proteins. The reduction of Pd (II) can be enhanced through the presence of endogenous electron mediators such as the menaquinone produced by the qoxB protein and flavin mononucleotide (FMN) produced by ssuE and ssuD. In addition, the ribosome-relevant proteins can maintain the translation, protein metabolism, and RNA process. There are upregulated flagellar motility, ATP-related proteins, and pentose-phosphate-pathway-related proteins, which can generate more energy to maintain normal metabolism, benefit-tending, and harm avoidance. These processes are conducive to enhancing the reduction of Pd (II). In this study, the mechanisms by which *B. megaterium* Y-4 produces biogenic Pd-NPs were elucidated through analyzing proteomics under two conditions.

## 2. Materials and Methods

### 2.1. Materials

TMT^®^ mass tagging kits and reagents were purchased from Thermo (Shanghai, China). A Bradford protein quantification kit was purchased from Beyotime (Shanghai, China). Dithiothreitol (DTT), iodoacetamide (IAM), ammonium bicarbonate, ammonium hydrogen triethylcarbate buffer (TEAB), ammonia solution, and trifluoroacetic acid (TFA) were purchased from Sigma. Sodium dodecyl sulfate (SDS) and carbamide were purchased from National Pharmaceutical Holdings Limited. Mass spectrometry grade pancreatic enzyme was purchased from Promega. LC-MS-grade ultrapure water and LC-MS-grade acetonitrile were purchased from Thermo Fisher Chemical (Shanghai, China). LC-MS-grade formic acid was purchased from Thermo Fisher Scientific (Shanghai, China), and acetone was purchased from Beijing Chemical Factory. A ProteoMiner low-abundance protein enrichment kit was purchased from Bio-Rad (Shanghai, China). To prepare the Pd (II) solution, Na_2_PdCl_4_ (Aladdin Industrial Corporation, Shanghai, China) was dissolved in distilled water.

### 2.2. Synthesis and Analysis of Microbial Pd (0) Nanoparticle

The *B. megaterium* Y-4 (MH472619) used in this study was isolated from the electronics waste factory. *B. megaterium* Y-4 was activated by inclined cultivation for 1–2 days before it was aerobically cultured in 100 mL Luria Broth medium in a 250 mL conical flask at 30 °C and 150 rpm. The log-growth *B. megaterium* Y-4 cells were obtained through centrifugation (4000 g, 5 min) and washed with 10 mL phosphate buffer (pH = 7.0) to remove the supernatant medium. Cells with 303 mg·L^−1^ (dry weight) were mixed with 10 mg·L^−1^ Pd (II) under N_2_ and N_2_-SF conditions, respectively. Then, *B. megaterium* Y-4 cells loaded with Pd-NPs were washed with ultrapure water and subjected to proteomics analysis. The detailed proteomics analysis procedures are described in the Supplementary Information (SI). The mass spectrometry proteomics data were deposited into the ProteomeXchange Consortium via the PRIDE [21] partner repository with the dataset identifier PXD049711. TMT was used for protein labeling and quantification (Table 1).

The residual Pd concentration was determined with an inductively coupled plasma mass spectrometer (ICP-MS, Agilent 7900, Santa Clara, CA, USA). All samples were filtered through 0.22 μm glass fiber filters (Tianjin Branch billion Lung Experimental Equipment Co., Ltd., Tianjin, China) before analysis. Ionic palladium (Pd) standard solutions (1000 mg·L^−1^) were diluted as the calibration solution and internal standard.

The removal efficiency (%) of Pd (II) was calculated by the following formula:

The removal efficiency (%) = (1 − *C*_e_/*C*_0_) × 100 (1)

where *C*_e_ (mg/L) is the Pd (II) residual concentration after reaction and *C*_0_ (mg/L) is the initial Pd (II) concentration.

The microbial Pd-NPs were freeze-dried and characterized by X-ray photoelectron spectroscopy (XPS, PHI X-Tool, DE). The XPS spectra were recorded with the Al Ka line at 15 kV and 51 W. The binding energies were determined at 284.8 eV by referencing the C1s component due to carbon being bound only to carbon or hydrogen. The biological slices used for transmission electron microscopy (TEM) (TECNAI 10, PHILIPS, NED) analysis were prepared following standard protocols [12].

## 3. Results

### 3.1. Production of Pd-NPs by B. megaterium Y-4

**Figure 1:**
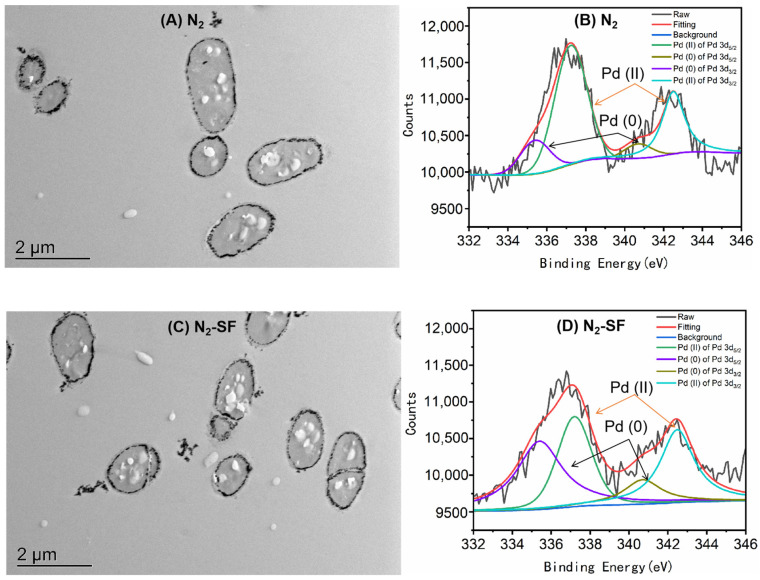
TEM and XPS of Pd-NPs synthesized by live *Bacillus megaterium* Y-4 under N_2_ (**A**,**B**) and N_2_-SF (**C**,**D**) conditions. Signals corresponding to Pd (II) and Pd (0) are indicated with arrows.

The bioreduction of palladium by *B. megaterium* Y-4 was investigated under anaerobic conditions with N_2_ and in presence of both N_2_ and 5 mM sodium formate (N_2_-SF). Sodium formate was used as an electron donor for the reduction. The removal efficiencies of Pd (II) reached 92.86% and 93.68% under N_2_ and N_2_-SF conditions, respectively (Figure S1). The chemical states of palladium after being treated with *B. megaterium* Y-4 under N_2_ and N_2_-SF conditions were further characterized by TEM and XPS spectroscopy. Firstly, the production of Pd-NPs by bacterial cells was observed: the TEM analysis demonstrated that many Pd-NPs were distributed in the periplasmic space and some Pd-NPs were also released from the live cells into the aqueous solution (Figure 1A,C). Secondly, the changes in the different states of the palladium were elucidated by XPS. As depicted in Figure 1B,D and Table S3, binding energies centered at 340.0–340.7 eV and 334.7–335.4 eV were observed, which was attributed to Pd 3d/2 and Pd 5d/2 of Pd (0). In addition, binding energies centered at 342.3–343.4 eV and 337.1–338.1 eV were observed, which was ascribed to Pd 3d/2 and Pd 5d/2 of Pd (II) [18]. The presence of Pd (0) after *B. megaterium* Y treatment demonstrates the conversion of palladium under the experimental conditions. Lastly, the quantification of the states of the palladium was achieved in the study and the conversion rates of the palladium under different conditions were compared. The XPS analysis (Table S3) indicated that the total peak area of Pd (0) synthesized by live cells was larger under N_2_-SF conditions (Figure 1D) than that under N_2_ conditions (Figure 1B). These results indicated that *B. megaterium* Y-4 can use the energy storage compounds to reduce Pd (II), and the exogenous electron donor sodium formate can further enhance the reduction of Pd (II) under N_2_ conditions.

### 3.2. Protein Identification and Comparison during Palladium Reduction

The protein levels of *B. megaterium* Y-4 were evaluated during palladium reduction to identify proteins that are critical for the reaction. Up to 2560 proteins from *B. megaterium* Y-4 were identified by proteomics analysis, and the parent ion mass tolerance distribution and principal component analysis are shown in Figures S2 and S3. The protein levels before and after treatment were comparted to obtain quantitative ratios and to understand the mechanisms for palladium reduction by this bacterium. A quantitative ratio greater than 1.2 was defined as upregulation, whereas a value below 0.833 was considered downregulation. All quantifiable proteins exhibiting increased (≥1.2-fold) and decreased (≤0.833-fold) expression levels are listed in Table S4 (*p* < 0.05).

**Figure 2:**
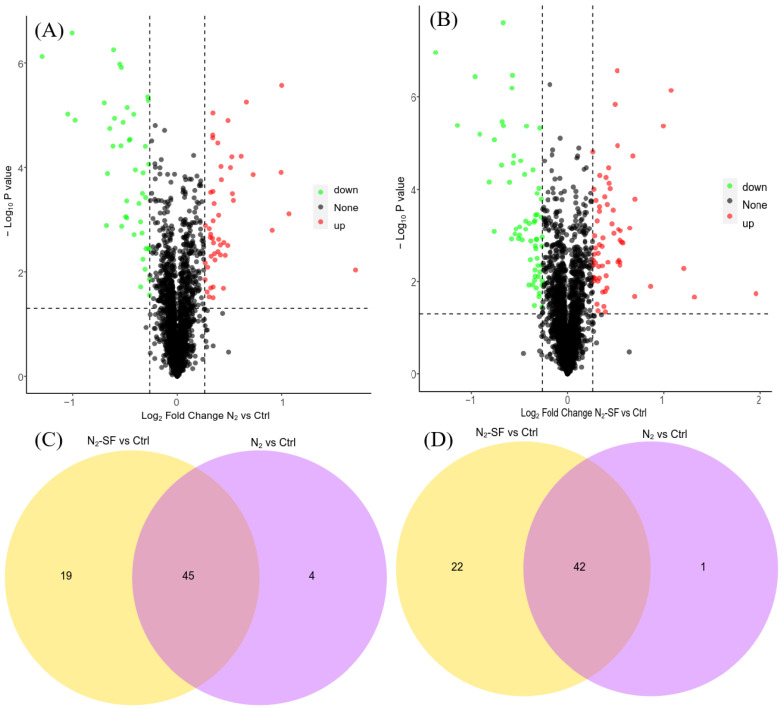
Volcano plots of DEPs in N_2_- (**A**) and N_2_-SF (**B**)-treated samples; Venn diagram showing the common and unique upregulated (**C**) or downregulated (**D**) expressed proteins. Upregulated and downregulated samples are indicated in yellow and purple, respectively.

Compared with the control sample without treatment, 49 upregulated proteins and 43 downregulated proteins in *B. megaterium* Y-4 were induced after treatment with N_2_ (Figure 2A and Figure S4A), 64 upregulated proteins and 64 downregulated proteins were identified after N_2_-SF treatment (Figure 2B and Figure S4A), and 45 upregulated proteins and 42 downregulated proteins were observed in samples treated under both conditions (Figure 2C,D and Figure S4B). Among these upregulated proteins, nirB (WP_098786927.1) exhibited the highest level of upregulation. Other upregulated proteins included hxlA (WP_013055599.1), spxA (WP_013055422.1), rpsO (WP_013058831.1), hfq (WP_013058791.1), galU (WP_161515009.1), and dinG (WP_013056100.1). The downregulated proteins included aceA (WP_013055343.1), katA (WP_098534856.1), trpB (WP_013059002.1), trpE (WP_013059006.1), trpA (WP_013059001.1), and trpC (WP_013059004.1) (Table S4). For the N_2_-treated sample, four upregulated proteins (WP_013056022.1, WP_013059402.1, WP_025752035.1, and cyoC (WP_053487176.1)) and one downregulated protein (WP_053487046.1) were unique and not observed in the N_2_-SF-treated sample (Figure 2C,D and Figure S4B). For the N_2_-SF-treated sample, 19 upregulated proteins (such as aroQ (WP_013059157.1), flgL (WP_057275290.1), panD (WP_013056099.1), and zwf (WP_013057959.1)) and 22 downregulated proteins (such as rpmI (WP_013059426.1, rpmB (WP_013058914.1), rpmG (WP_013054911.1), and qoxB (WP_013056572.1)) were unique (Figure 2C,D and Figure S4B). Nearly all of the DEPs exhibited either direct or in direct interactions with other proteins (Figures S5 and S6).

### 3.3. Functional Characterization of Differentially Expressed Proteins

These DEPs under different conditions were classified according to their subcellular locations (Figure S7). For the N_2_-treated sample, 47.37%, 26.32%, 15.79%, and 10.53% of the DEPs were located in the cytoplasm, cell membrane, extracell, and cell wall, respectively. For the N_2_-SF-treated sample, 41.67%, 33.33%, 16.67%, and 8.33% of the DEPs were located in the cytoplasm, cell membrane, extracell, and cell wall, respectively.

**Figure 3:**
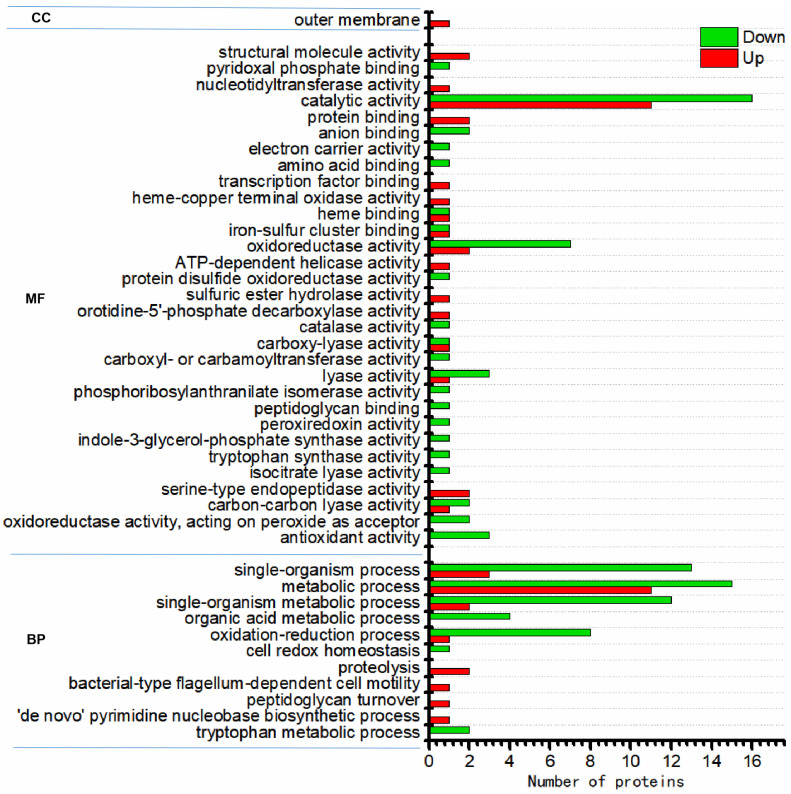
Functional category distribution (CC, cellular component; MF, molecular function; BP, biological process) of differentially expressed proteins in the N_2_-treated sample.

**Figure 4:**
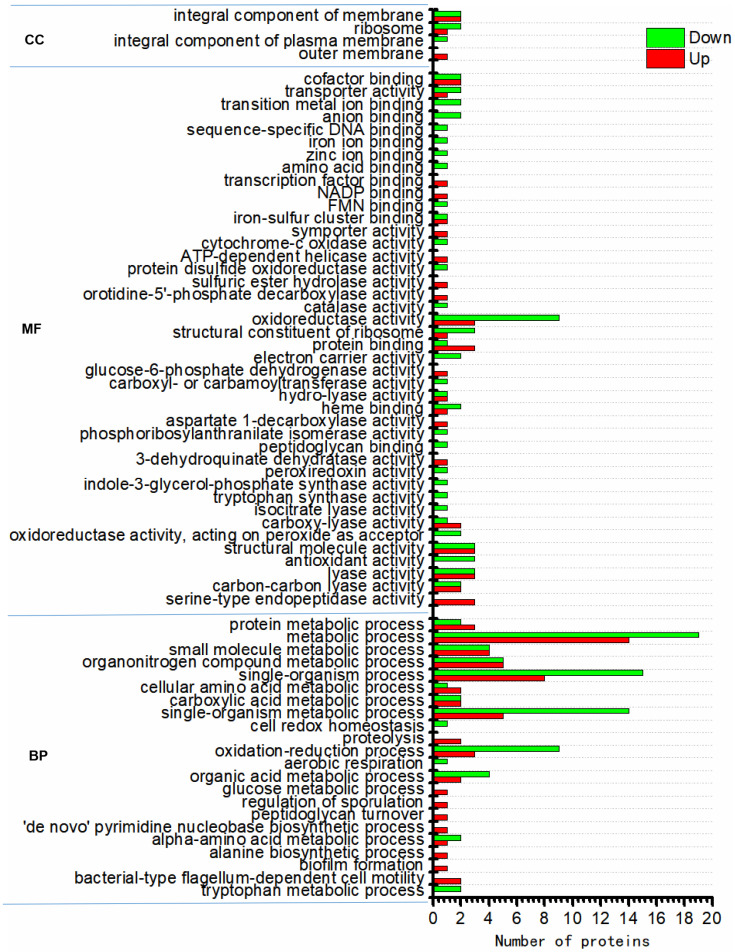
Functional category distribution (CC, cellular component; MF, molecular function; BP, biological process) of differentially expressed proteins in the N_2_-SF-treated sample.

Based on the Gene Ontology (GO) analysis tool, the biological functions affected by DEPs were classified to three functional groups, namely, molecular function (MF), cellular component (CC), and biological process (BP). For the N_2_-treated sample, the order of the number of these DEPs enriched in GO functions was as follows: catalytic activity (GO:000382) (11 upregulated and 16 downregulated proteins) > metabolic process (GO:0008152) (11 upregulated and 15 downregulated proteins) > single-organism process (GO:0044699) (3 upregulated and 13 downregulated proteins) > single-organism metabolic process (GO:0044710) (2 upregulated and 12 downregulated proteins) > oxidation-reduction process (GO:0055114) (1 upregulated and 8 downregulated proteins) > oxidoreductase activity (GO:0016491) (2 upregulated and 7 downregulated proteins) (Figure 3). For the N_2_-SF-treated sample, the order of the number of these DEPs enriched in GO functions was as follows: metabolic process (GO:0008152) (14 upregulated and 19 downregulated proteins) > single-organism process (GO:0044699) (8 upregulated and 15 downregulated proteins) > single-organism metabolic process (GO:0044710) (5 upregulated and 14 downregulated proteins) > oxidation-reduction process (GO:0055114) (3 upregulated proteins and 9 downregulated proteins) > oxidoreductase activity (GO:0016491) (3 upregulated and 9 downregulated proteins) > organonitrogen compound metabolic process (GO:1901564) (5 upregulated and 5 downregulated proteins) > small molecule metabolic process (GO:0044281) (4 upregulated and 4 downregulated proteins) (Figure 4).

### 3.4. Functional Enrichment of Differentially Regulated Proteins

**Figure 5:**
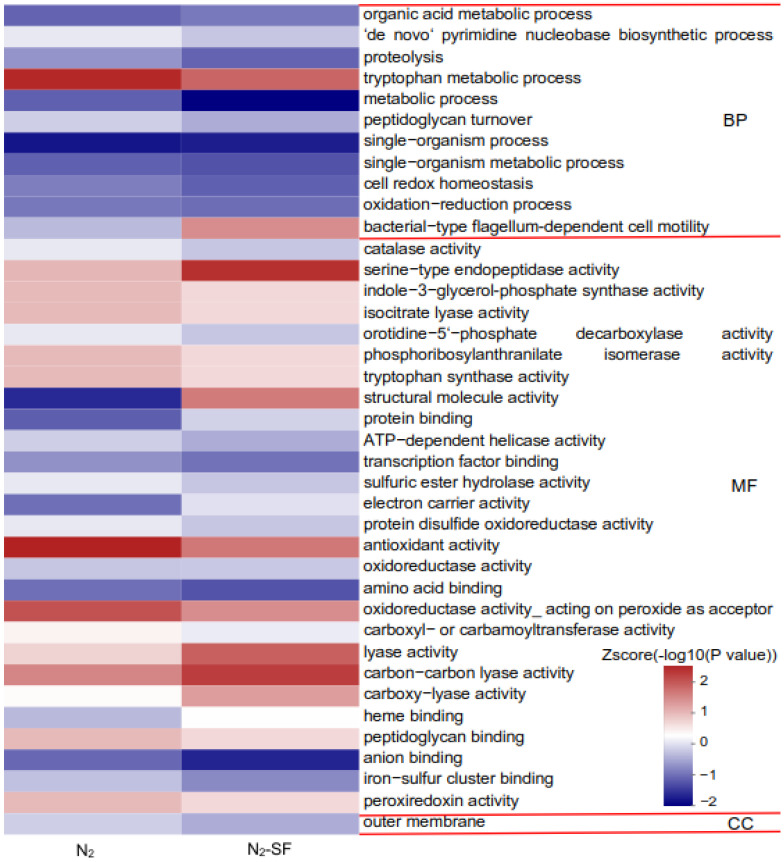
Function of the affected proteins during palladium reduction. A hierarchical clustering analysis was conducted on the differentially expressed proteins based on GO-analysis-based enrichment. The *p*-values were converted into Z-scores, which are displayed in the color legend with a red hue indicating significant enrichment. CC, cellular component; MF, molecular function; BP, biological process.

To investigate the functional differences in the upregulated and downregulated proteins, these proteins were subjected separately for GO, KEGG pathway, and domain enrichment analysis. The GO enrichment analysis results are shown in Figure 5, Figures S8 and S9. For the N_2_-treated sample, the upregulated proteins were mainly enriched in serine-type endopeptidase activity (GO:0004252), hydrolase activity (GO:0016787), orotidine-5′-phosphate decarboxylase activity (GO:0004590), sulfuric ester hydrolase activity (GO:0008484), and the ‘de novo’ pyrimidine nucleobase biosynthetic process (GO:0006207) (Figure S8A). Meanwhile, the downregulated proteins were mainly related to antioxidant activity (GO:0016209), the tryptophan metabolic process (GO:0006568), oxidoreductase activity (GO:0016684), the single-organism metabolic process (GO:0044710), and the oxidation-reduction process (GO:0055114) (Figure S8B). For the N_2_-SF-treated sample, the upregulated proteins were mainly involved in serine-type endopeptidase activity (GO:0004252), bacterial-type flagellum-dependent cell motility (GO:0071973), 3-dehydroquinate dehydratase activity (GO:0003855), biofilm formation (GO:0042710), aspartate 1-decarboxylase activity (GO:0004068), the alanine biosynthetic process (GO:0006523), carboxy-lyase activity (GO:0016831), and glucose-6-phosphate dehydrogenase activity (GO:0004345) (Figure S9A). The downregulated proteins mainly significantly participated in antioxidant activity (GO:0016209), the tryptophan metabolic process (GO:0006568), oxidoreductase activity (GO:0016684), the single-organism metabolic process (GO:0004451), and tryptophan synthase activity (GO:0004834) (Figure S9B).

**Figure 6:**
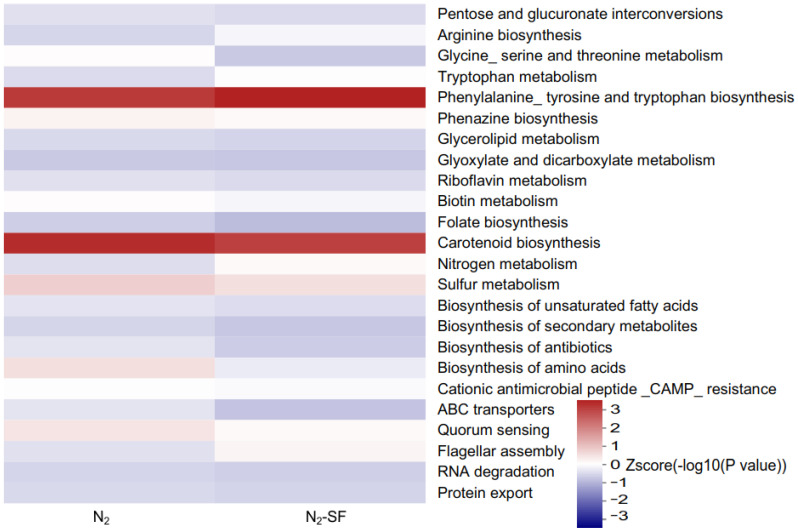
Affected pathways during palladium reduction. KEGG-pathway-based enrichment was employed to conduct a hierarchical clustering analysis on the proteins that were expressed differently. The *p*-values were transformed into Z-scores for hierarchical clustering analysis. The Z-score is shown in the color legend, and the red color represents significant enrichments.

The KEGG pathway enrichment results are shown in Figure 6, Figures S10 and S11. For the N_2_-treated sample, the upregulated proteins were responsible for quorum sensing (map02024) and cationic antimicrobial peptide (CAMP) resistance (map01503) (Figure S10A); the downregulated proteins were critical for phenylalanine, tyrosine, and tryptophan biosynthesis (map00400), carotenoid biosynthesis (map00906), sulfur metabolism (map00920), the biosynthesis of amino acids (map01230), the biosynthesis of secondary metabolites (map01110), ABC transporters (map02010), and phenazine biosynthesis (map00405) (Figure S10B). For the N_2_-SF-treated sample, the upregulated DEPs were important for flagellar assembly (map02040), nitrogen metabolism (map00910), and quorum sensing (map02024) (Figure S11B); the downregulated DEPs were important for phenylalanine, tyrosine, and tryptophan biosynthesis (map00400), carotenoid biosynthesis (map00906), and sulfur metabolism (map00920) (Figure S11B).

**Figure 7:**
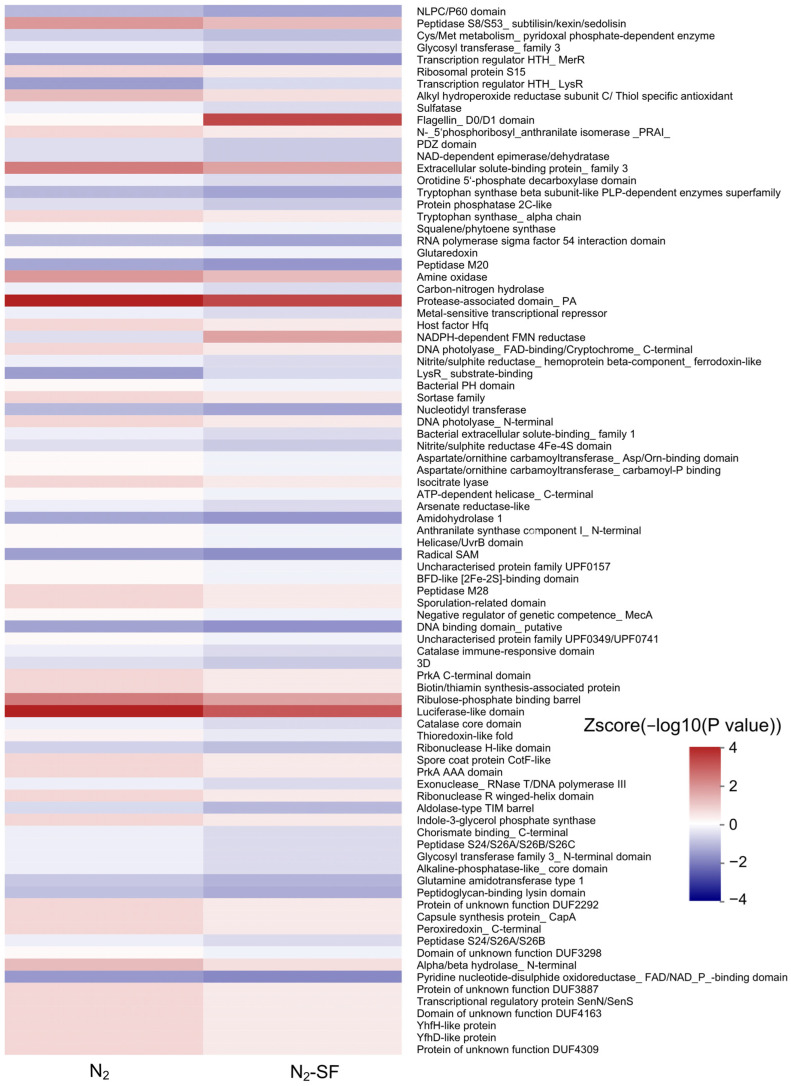
Hierarchical clustering analysis was conducted for the differentially expressed proteins according to protein-domain-based enrichment. The *p*-values were transformed into Z-scores for hierarchical clustering analysis. The Z-score is shown in the color legend, and the red color represents significant enrichments.

The DEPs in all cases were also examined using protein domain enrichment analysis (Figure 7, Figures S12 and S13). For the N_2_-treated sample, the upregulated proteins included protease-associated domain (IPR003137), peptidase S8/S53, subtilisin/kexin/sedolisin (IPR000209), alpha/beta hydrolase, N-terminal (IPR022742), and ribosomal protein S15 (IPR000589) (Figure S12A). The downregulated expressed proteins contained luciferase-like domain (IPR011251), extracellular solute-binding protein, family 3 (IPR001638), amine oxidase (IPR002937), alkyl hydroperoxide reductase subunit C/Thiol-specific antioxidant (IPR000866), and thioredoxin-like fold (IPR012336) (Figure S12B). For the N_2_-SF-treated sample, the upregulated proteins included flagellin, D0/D1 domain (IPR001029), protease-associated domain, PA (IPR003137), peptidase S8/S53, subtilisin/kexin/sedolisin (IPR000209), and alpha/beta hydrolase, N-terminal (IPR022742) (Figure S13A); the downregulated proteins included luciferase-like domain (IPR011251), extracellular solute-binding protein, family 3 (IPR001638), NADPH-dependent FMN reductase (IPR005025), amine oxidase (IPR002937), and alkyl hydroperoxide reductase subunit C/Thiol-specific antioxidant (IPR000866) (Figure S13B).

### 3.5. Metabolic Pathways Identified for Palladium Reduction

**Figure 8:**
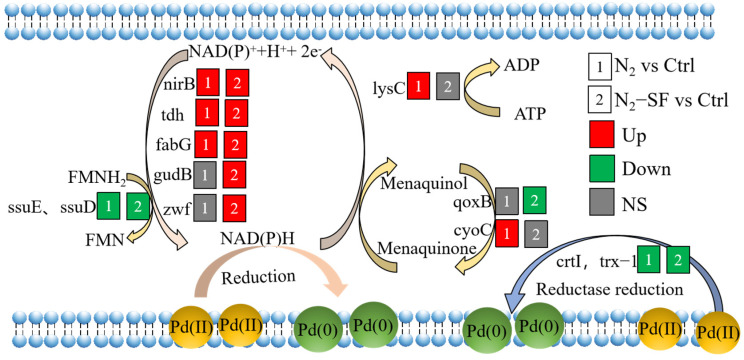
Proposed reduction mechanism of Pd (II) by *Bacillus megaterium* Y-4. Colored boxes indicate the differential expression profiles (upregulated, red; downregulated, green; no significance (NS), gray) for the reduction of Pd (II) by *B. megaterium* Y-4 cells under anaerobic conditions in the presence of N_2_ (1), and both N_2_ and 5 mM sodium formate (N_2_-SF) (2) compared with pure cells without palladium. The information of genes involved in these cellular metabolisms is shown in Supplementary Table S4.

Based on the above proteome results, several metabolic pathways of palladium bioreduction by *B. megaterium* Y-4 were proposed (Figure 8, Table S4). Firstly, NAD(P)H with strong reducibility can directly reduce Pd (II) [22,23]. In *B. megaterium* Y-4, the NAD(P)H-generating proteins included nirB, tdh, fabG, gudB, zwf, and ssuE. For the N_2_-SF-treated sample, only ssuE was downregulated, while nirB, tdh, fabG, gudB, and zwf were upregulated. For the N_2_-treated sample, the following expression pattern was observed: only ssuE downregulated, gudB and zwf nonsignificant, and nirB, tdh, and fabG upregulated. Furthermore, the expression of tdh and fabG in the N_2_-SF-treated sample was higher than that in the N_2_-treated sample, which was consistent with the trend of the Pd (II) reduction amount. Figure S14 and Table S4 summarize the metabolic pathways involved by some proteins. The nirB and gudB proteins were involved in nitrogen metabolism (map00910) (Figure S14A). The fabG (WP_098239123.1) protein played a role in biotin metabolism (map00780) (Figure S14B). The ssuE protein was critical for riboflavin metabolism (map00740) (Figure S14C). The reduction of Pd (II) may be achieved through these metabolic processes. A study showed that NADH dehydrogenases of *Shewanella oneidensis* MR-1 that can produce NADH contribute to the reduction of Pd (II) [17]. The NADH also contributes to Se (VI) bioreduction through regulating the electron transfer [24]. Secondly, reductase plays significant roles in a series of intracellular redox reactions and is involved in various electron transfer cycles. In the N_2_-SF-treated sample, four reductase proteins (trx-1, flavin reductase family protein, and two crtIs) were downregulated (Table S4). In the N_2_-treated sample, three proteins (trx-1 and two crtIs) were downregulated (Table S4). Pd (II) may be reduced as electron acceptors under the action of these reductases. The crtI proteins may reduce Pd (II) through carotenoid biosynthesis (map00906) and the biosynthesis of secondary metabolites (map01110) (Table S4). Thirdly, previous studies have reported that Q-circle [25] and riboflavin synthase [26] can act as endogenous electron mediators and accelerate the electron transfer process in the bacterial cells, enhancing the efficiency of Pd (II) bioreduction. For the N_2_-SF-treated samples, the qoxB Q-cycle protein and three riboflavin-related proteins (LLM-class flavin-dependent oxidoreductase, ssuD, ssuE) were downregulated. For the N_2_-treated samples, the cyoC Q-cycle protein was upregulated, and three riboflavin-related proteins (the LLM-class flavin-dependent oxidoreductase, ssuD and ssuE) were downregulated. The ssuD and ssuE proteins are significantly enriched in sulfur metabolism (map00920) (Table S4). These results suggested that *B. megaterium* Y-4 may defend Pd (II) stress by reducing sulfate uptake. Chromate also as a competitive inhibitor of sulfate uptake [27]. Fourthly, The DinG, lysC and ATP-binding cassette domain-containing protein can generate energy to maintain normal metabolism for reduction of Pd (II). For the N_2_-treated sample, the DinG and lysC were both up-regulated. The lysC proteins was significantly enriched in glycine, serine and threonine metabolism (map00260) (Figure S15). In the N_2_-SF-treated sample, the DinG- and ATP-binding cassette-domain-containing proteins were both upregulated. ABC transporters are transmembrane proteins that utilize energy to carry substrates into the cells [28]. The N_2_-treated sample and N_2_-SF-treated sample exhibited a decrease in the transporter substrate-binding-domain-containing protein, amino acid ABC transporter substrate-binding protein, and extracellular solute-binding protein. The iron–hydroxamate ABC transporter substrate-binding protein was also downregulated in the N_2_-treated sample. These results indicate that the energy generated can be increased and the transport proteins are inhibited under N_2_ and N_2_-SF conditions. Fifthly, the ribosome-relevant proteins (e.g., rpsO, rpmG, rpmB, and GNAT family N-acetyltransferase) participate in translation, protein metabolism, and RNA processes. As a consequence, more proteins are synthesized to maintain the cellular structure and function, to repair damaged proteins, and to produce new proteins against Pd (II) stress. In the N_2_-treated sample, the rpsO was upregulated and the GNAT family N-acetyltransferase was downregulated. For the N_2_-SF-treated sample, the rpsO was upregulated while the GNAT family N-acetyltransferase, rpmG, and rpmB were downregulated. Lastly, flagellar motility is very important to allow bacteria to move toward favorable conditions, form biofilms, and acquire nutrients [29,30]. *B. megaterium* Y-4 could exhibit benefit-tending and harm-avoiding behaviors through the flagellum and motility under Pd (II) stress. The fliC (FC = 2.1) was upregulated in the N_2_-treated sample. The FlgL (FC = 1.22) and fliC (FC = 2.3) were upregulated in the N_2_-SF-treated sample. The fliC protein was significantly enriched in flagellar assembly (map02040).

## 4. Discussion

The recycling of palladium is an important task due to its importance in industry and potential harm to the environment. The microbial reduction of palladium has been used in treating palladium-containing water and it is an economic and green strategy that does not pollute the environment. As metal reduction is a complicated process, many proteins are involved in this process. Understanding the pathways of proteins during palladium reduction will provide valuable insights into more efficient processing and provide useful information for understanding the response of a bacterium under stress. In this study, we used *B. megaterium* Y-4 to reduce palladium under anaerobic conditions in the absence and presence of sodium formate. The palladium removal efficiency can be improved in the presence of an external electron donor. The Pd nanoparticles were observed both inside and outside of the bacterial cells (Figure 1). Our XPS experiment also confirmed the reduction of palladium by *B. megaterium* Y-4.

Proteomics analysis was employed to explore the changes in the protein levels in the bacterium during palladium reduction. A number of proteins were identified (Figure 2), which are critical for understanding the pathways that are critical for palladium reduction. The locations of the DEPs include the cell wall, extracell, cell membrane, and cytoplasm, which suggests that the microbial reduction of palladium in this study may include the transportation of palladium across the cell wall into the cytoplasm, which requires proteins at these locations. The upregulation of proteins suggests that palladium may stimulate certain pathways to reduce the toxic effect caused by metal taking. The downregulation of proteins might arise from the toxic effect of palladium. Nonetheless, based on GO and KEGG analysis, the following pathways were affected during the microbial reduction of palladium. NAD(P)H may play important roles in palladium reduction as several related proteins were upregulated [17]. Interestingly, the downregulation of reductase and oxidoreductase was observed under both conditions, which suggests that these enzymes play important roles in the reduction reaction [19], while the lower protein level might be due to their involvement in reduction and palladium did not induce their production. In addition, proteins related to energy synthesis were stimulated and the levels of proteins for transportation were reduced. The results suggest that the uptake of palladium may result in the denature of some transportation proteins and a reduction in the required additional energy, which may contribute to the restoration of the function of proteins [28], the response to the toxic effect of the metal, and the synthesis of more proteins. Indeed, some ribosome-related proteins were also affected during palladium reduction. The Dep data also showed that palladium also affected the motion of the bacterium, as proteins related to flagellar motility such as fliC were upregulated.

Based on the data accumulated in this study, *B. megaterium* Y-4 may utilize endogenous electron donors (such as NAD(P)H) or reductase to provide electrons for Pd (II) reduction. Endogenous electron mediators (quinones and flavins) may further enhance Pd (II) reduction [18,25]. The ribosome-relevant proteins can maintain the translation, protein metabolism, and RNA process. More energy is required to maintain metabolisms, benefit-tending, and harm avoidance through the ATP-related protein, flagellum motility, and pentose phosphate pathway. The identification of these proteins demonstrated that palladium reduction is a complicated process involving multiple enzymes and proteins in several pathways. Therefore, our study provides insights into the mechanism of palladium reduction in *B. megaterium* Y-4. Based on the obtained information, further study on individual proteins or pathways will expand our knowledge on the function of these proteins. The information is also helpful for improving metal removal efficiency in microbial treatment.

## 5. Conclusions

In summary, the microbial reduction of Pd (II) to Pd-NPs by *B. megaterium* Y-4 can be achieved through the synergistic action of multiple proteins involved in several cellular pathways. We found that NirB, tdh, fabG, gudB, and zwf proteins produce NAD(P)H, which can provide electrons for *B. megaterium* Y-4 for the reduction of Pd (II). Additionally, Pd (II) may also be reduced by the reductase produced by the ssuE, trx-1, and flavin reductase family protein. The reduction efficiency can be accelerated through the menaquinone produced by the qoxB protein and FMN produced by ssuE and ssuD. Further investigations are still needed on how these genes or proteins influence the microbial reduction of Pd (II) in future work. Our findings provide a new perspective for understanding the bioreduction mechanisms of Pd (II) to create Pd-NPs.

## Figures and Tables

**Table 1:** Labeling information of the sample.

Run Name	126	127N	128N	129N	130N	131N	132N	133N	134N
run1	Ctrl-1	Ctrl-2	Ctrl-3	N_2_-1	N_2_-2	N_2_-3	N_2_-SF-1	N_2_-SF-2	N_2_-SF-3

## Data Availability

Data available on reasonable request.

## Supplementary Materials

The following supporting information can be downloaded at: https://www.mdpi.com/article/10.3390/nano14060512/s1, Table S1. peptide fraction separation liquid chromatography elution gradient table. Table S2. Liquid chromatography elution gradient table. Table S3. The Pd (II) and Pd (0) XPS peak area of different samples. Table S4. All the identified quantifiable upregulated and downregulated proteins. Figure S1. The removal efficiency (%) of Pd (II) by *B. megaterium* Y-4 under N_2_, and N_2_-5 mM sodium formate (N_2_-SF) conditions, respectively. Figure S2. Parent ion mass tolerance distribution. The abscissa is the mass deviation, and the ordinate is the parent ion density distribution of the corresponding error. Most peaks close to 0, suggesting small mass deviation. Figure S3. Principal component analysis. The horizontal axis PC1 and vertical axis PC2 represent the scores of the first and second ranked principal components, respectively, and the scatter color represents the experimental grouping of the samples. Figure S4. Number of differentially expressed proteins (A) and venn diagram showing common and unique differentially expressed proteins were compared (B). The DEPs under different conditions are shown in different color. The proteins were observed under both conditions are shown, and 87 proteins were present under both conditions. Figure S5. The protein and protein interaction networks of all differentially expressed proteins in N_2_ treated sample. Up-(red) and down-(green) regulated DEPs are indicated. Size of the dot correlates to the absolute value of N_2_ treated log2FC. Figure S6. The protein and protein interaction networks of all differentially expressed proteins in the N_2_-SF treated sample. Up-(red) and down-(green) regulated DEPs are shown. Size of the dot corelates to the absolute value of N_2_-SF treated log2FC. Figure S7. Subcellular location of differentially expressed proteins. N_2_ and N_2_-SF treated samples were analyzed and the proteins from different locations are shown in different color. Figure S8. GO-based enrichment analysis of up-regulated proteins (A, red) and down-regulated proteins (B, green) in N_2_ treated sample. Figure S9. GO-based enrichment analysis of up-regulated (A, red) and down-regulated (B, green) proteins in the N_2_-SF treated sample. Figure S10. KEGG-based enrichment analysis of up-regulated (A, red) and down-regulated (B, green) proteins of N_2_ treated sample. Figure S11. KEGG-based enrichment analysis of up-regulated (A, red) and down-regulated (B, green) proteins of N_2_-SF treated sample. Figure S12. Protein domain-based enrichment analysis of up-regulated (A, red) and down-regulated (B, green) proteins of N_2_ treated sample. The protein domains are indicated on the left side and the possibility values are indicated on the right side. Figure S13. Protein domain-based enrichment analysis of up-regulated (A, red) and down-regulated (B, green) proteins of N_2_-SF treated sample. The protein domains are indicated on the left side and the possibility values are indicated on the right side. Figure S14. Schematic diagram of nitrogen metabolism, biotin metabolism and riboflavin metabolism. Colored boxes indicate the different expression profiles (upregulated, red; downregulated, green; no significance (NS), gray) under N_2_ (1) and N_2_-SF (2) conditions compared with cells without exposing to palladium. The information of upregulated or downregulated proteins involved in these cellular metabolisms is shown in the Supplementary Table S3. Figure S15. Schematic diagram of glycine, serine and threonine metabolism. Colored boxes indicate the different expression profiles. Upregulated, downregulated and no significantly (NS) changed pathways are shown in red, green and gray, respectively under N_2_ (1) and N_2_-SF (2) conditions compared with cells without exposing to palladium. The information of upregulated or downregulated proteins involved in these cellular metabolisms is shown in the Supplementary Table S4. References [31,32,33,34,35,36] are cited in the Supplementary Materials.
