# Supplementary material for: The Pd (II) Reduction Mechanisms in Bacillus megaterium Y-4 Revealed by Proteomic Analysis
Source: Nanomaterials (Basel). 2024 Mar 12;14(6):512. doi: 10.3390/nano14060512 (PMC10976171; doi:10.3390/nano14060512)
Supplement: Supplementary file 1 [file nanomaterials-14-00512-s001.zip › nanomaterials-2856161-supplementary.pdf]

Supplementary materials

The Pd (II) Reduction Mechanisms in *Bacillus megaterium* Y-4 Revealed by Proteomic Analysis

Yuan Chen [1,2], Jiaxing Wang [3], Daidi Chen [1], Boxi Wang [1], Jin Chuan Wu [1], Rongrong Liu [2,*] and Qingxin Li [1,*]

1 Guangdong Provincial Engineering Laboratory of Biomass High Value Utilization, Institute of Biological and Medical Engineering, Guangdong Academy of Sciences, Guangzhou 510316, China; chyuan0019@163.com (Y.C.); chendd1591@163.com (D.C.); bessycc@163.com (B.W.); wu_jinchuan@outlook.com (J.C.W.)

2 Key Laboratory for Water Quality and Conservation of the Pearl River Delta, Institute of Environmental Research at Greater Bay Area, Ministry of Education, Guangzhou University, Guangzhou 510006, China

3 Ministry of Education Key Laboratory of Pollution Control and Ecological Remediation for Industrial Agglomeration Area, School of Environment and Energy, South China University of Technology, Guangzhou 510006, China; 19120332369@163.com

* Correspondence: liurr@gzhu.edu.cn (R.L.); qingxin_li@outlook.com (Q.L.)

S1. Proteomics analysis methods

1.1 Protein extraction[1-2]

The bacterial cells were transferred to a 1.5 mL centrifuge tube and lysed with SDT lysis buffer (300 μL) containing 4% SDS, 10 mM DL-Dithiothreitol (DTT), and 100 mM triethylammonium bicarbonate (TEAB), followed by 5 min of ultrasonication in an ice bath using ultrasonic cell breaker (JY92-11N). The lysate was centrifuged at 12,000 g for 15 min at 4°C and the supernatant was reduced with 10 mM DTT for 1 h at 56°C, and subsequently alkylated with iodoacetamide (30 μL, 0.5 M) for 1 h at room temperature in the dark. Then samples were mixed thoroughly with 4 times volume of precooled acetone by vortexing with turbine mixer (HY-6B) and incubated at -20°C for at least 2 h. Samples were then centrifuged at 12,000 g for 15 min at 4°C and the precipitation was collected. After washing with 1 mL cold acetone, the pellet was dissolved in dissolution buffer (200 μL) that contained 8 M Urea, 100 mM TEAB and pH 8.5.

1.2 Protein Quantification

BSA standard protein solution was prepared according to the instructions of Bradford protein quantitative kit, with gradient concentration ranged from 0 to 0.5 $g \cdot L^{-1}$. BSA standard protein solutions and sample solutions with different dilution multiples were added into a 96-well plate to a final volume to 20 μL, respectively. Each gradient was repeated three times. The plate was added 180 μL G250 dye solution quickly and placed at room temperature for 5 min. The absorbance at 595 nm was measured with Microplate reader (Thermo/Multiskan FC). The standard curve was obtained by plotting the absorbance of standard protein solution against the protein concentration of the sample. 20 μg of the protein sample was loaded into a 12% SDS-PAGE gel for electrophoresis, wherein the stacking gel was performed at 80 V for 20 min, and the separation gel was performed at 120 V for 90 min. The gel was stained by Coomassie brilliant blue R-250 and destained until the bands were visualized clearly[3].

1.3 TMT Labeling of Peptides

Each protein sample was taken and the volume was made up to 100 μL with DB dissolution buffer that contained 8 M Urea, 100 mM TEAB and pH 8.5. Trypsin and 100 mM TEAB buffer were added and sample was mixed and digested at 37°C for 4 h. Trypsin and $CaCl_2$ were then added and resulting sample was kept overnight for digestion. Formic acid (FA) was mixed with digested sample to adjust pH to 3.0. The sample was centrifuged at 12,000 g for 5 min at room temperature. The supernatant was slowly loaded to the C18 desalting column, washed with washing buffer (700 μL) that contained 0.1% FA and 3% acetonitrile 3 times. The sample was then eluted with 200 μL elution buffer that contained 0.1% FA and 70% acetonitrile. The eluents of each sample were collected and lyophilized in freeze dryer (Labogene/ Scan Speed 40). 100 μL

of 0.1 M TEAB buffer was added into the samples to reconstitute, and 41 μL of acetonitrile-dissolved TMT labeling reagent was added. The sample was mixed through shaking for 2 h at room temperature. The reaction was stopped by addition of equal volume of 8% ammonia. All labeling samples were desalted and lyophilized.

1.4 Separation of fractions

Mobile phase solution A (2% acetonitrile, adjusted pH to 10.0 using ammonium hydroxide) and solution B (98% acetonitrile) were used to develop a gradient elution. The lyophilized powder was dissolved in solution A and centrifuged at 12,000 g for 10 min at room temperature. The sample was fractionated using a C18 column (Waters BEH C18, 4.6×250 mm, 5 μm) on a Rigol L3000 HPLC system in which the column oven was set as 45°C. The detail of elution gradient was shown in **Table S1**. The eluates were monitored at a wave length of 214 nm, collected for one tube per minute and combined into 10 fractions finally. All fractions were dried under vacuum, and then, reconstituted in 0.1% (v/v) FA in water.

Table S1. Peptide fraction separation liquid chromatography elution gradient table.

Time（min）	flow rate（$mL·min^{-1}$）	mobile phase solution A (%)	mobile phase solution B (%)
0	1	97	3
10	1	95	5
30	1	80	20
48	1	60	40
50	1	50	50
53	1	30	70
54	1	0	100

1.5 LC-MS/MS Analysis

For transition library construction, shotgun proteomics analyses were performed using an EASY-nLCTM 1200 UHPLC system (LC140, Thermo Fisher) coupled with a Q Exactive^{TM} HF-X mass spectrometer (Thermo Fisher) operating in the data-dependent acquisition (DDA) mode. 1 μg sample was injected into a home-made C18 Nano-Trap column (4.5 cm × 75 μm, 3 μm). Peptides were separated in a home-made analytical column (15 cm × 150 μm, 1.9 μm), using a linear gradient elution as listed in **Table S2**. The separated peptides were analyzed by Q Exactive^{TM} HF-X mass spectrometer (Thermo Fisher), with ion source of Nanospray Flex™（ESI）, spray voltage of 2.1 kV and ion transport capillary temperature of 320°C. Full scan ranges from *m/z* 350 to 1,500 with resolution of 60000 (at *m/z* 200). An automatic gain control (AGC) target value was 3×10^6 and a maximum ion injection time was 20 ms. The top 40 precursors of the highest abundant in the full scan were selected and fragmented by higher energy collisional dissociation (HCD) and analyzed in MS/MS, where resolution was 30,000 (at *m/z* 200) for 6 plex. The automatic gain control (AGC) target value was 5×10^4 and the maximum ion injection time was 54 ms. A normalized collision energy was set as 32%, an intensity threshold was 1.2×10^5, and the dynamic exclusion parameter was 20 s, respectively.

Table S2. Liquid chromatography elution gradient table.

Time (min)	flow rate (nL·min^{-1})	mobile phase A solution (%)	mobile phase B solution (%)
0	600	94	6
2	600	85	15
48	600	60	40
50	600	50	50

51	600	45	55
60	600	0	100

1.6 Data analysis

1.6.1 Protein identification and quantitation

The resulting spectra from each run were searched separately against *Bacillus*-*megaterium*-NCBI database by the search engines-Proteome Discoverer 2.4 (PD 2.4, Thermo). The searched parameters are set as follows: mass tolerance for precursor ion was 10 ppm and mass tolerance for product ion was 0.02 Da. Carbamidomethyl was specified as fixed modifications, Oxidation of methionine (M) and TMT plex were specified as dynamic modification. Acetylation, TMT plex, Met-loss and Met-loss+Acetyl were specified as N-Terminal modification in PD 2.4. A maximum of 2 missed cleavage sites were allowed.

In order to improve the quality of analysis results, the software PD 2.4 further filtered the retrieval results. Peptide spectrum matches (PSMs) with a credibility of more than 99% was identified PSMs. The identified protein contains at least 1 unique peptide. The identified PSMs and protein were retained and performed with false discovery rate less than 1.0%. The protein quantitation results were statistically analyzed by T-test. The proteins whose quantitation significantly different between experimental and control groups, ($p < 0.05$ and $FC \geq 1.2$ or $FC \leq 0.833$), were defined as differentially expressed proteins (DEP).

1.6.2 Functional analysis of DEP

Gene Ontology (GO) and InterPro (IPR) functional analysis were performed using the interproscan program against the non-redundant protein database including Pfam, PRINTS, ProDom, SMART, ProSite, and PANTHER[4]. Databases of COG (Clusters of Orthologous

Groups) and KEGG (Kyoto Encyclopedia of Genes and Genomes) were used to analyze the protein families and pathways. DEPs were used for Volcanic map analysis, cluster heat map analysis and enrichment analysis of GO, IPR and KEGG[5]. The protein-protein interactions were predicted using the STRING-db server[6] (http://string.embl.de/).

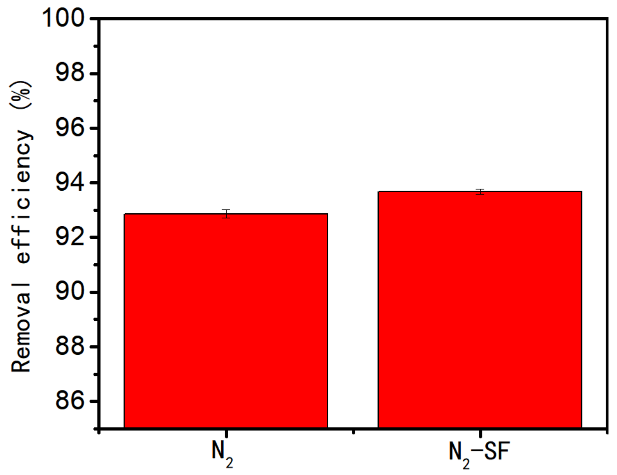

Figure S1. The removal efficiency (%) of Pd (II) by *B. megaterium* Y-4 under N_2, and N_2-5mM sodium formate (N_2-SF) conditions, respectively.

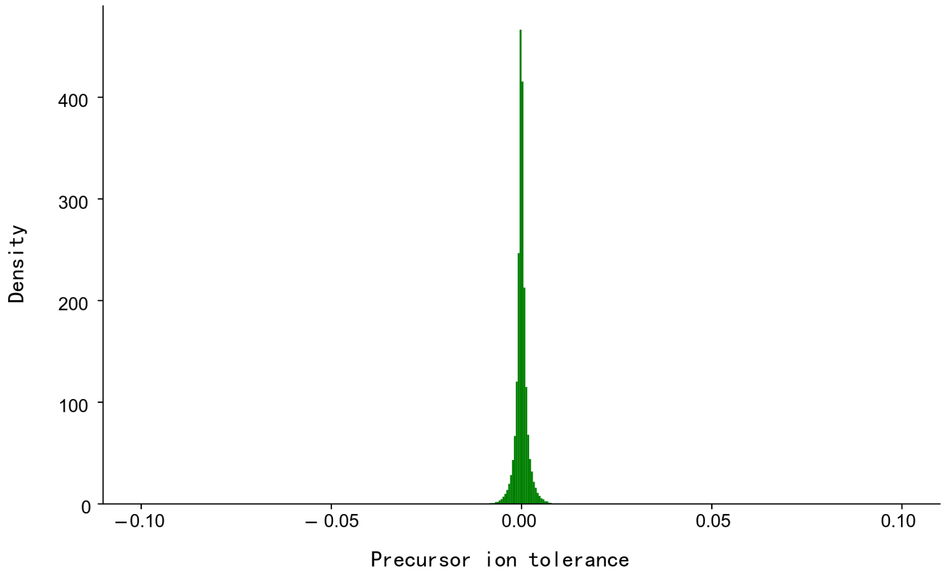

Figure S2. Parent ion mass tolerance distribution. The abscissa is the mass deviation, and the ordinate is the parent ion density distribution of the corresponding error. Most peaks close to 0, suggesting small mass deviation.

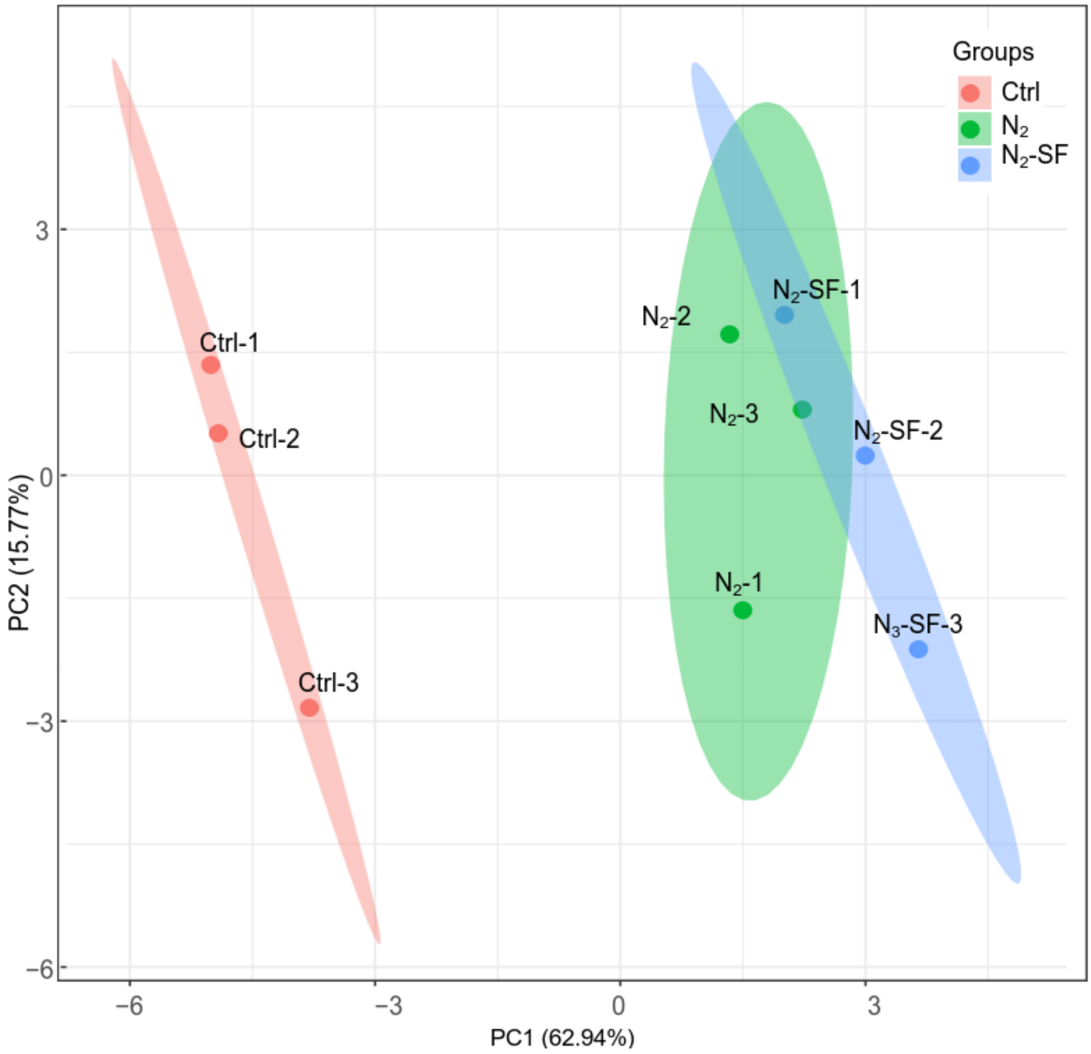

Figure S3. Principal component analysis. The horizontal axis PC1 and vertical axis PC2 represent the scores of the first and second ranked principal components, respectively, and the scatter color represents the experimental grouping of the samples.

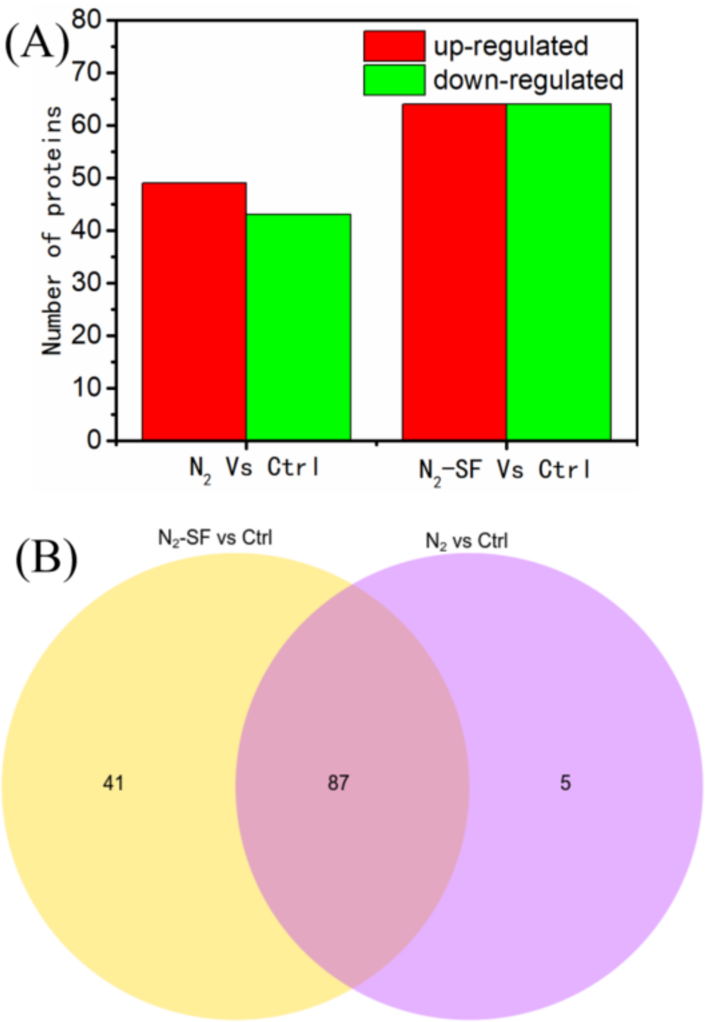

Figure S4. Number of differentially expressed proteins (A) and venn diagram showing common and unique differentially expressed proteins were compared (B). The DEPs under different conditions are shown in different color. The proteins were observed under both conditions are shown, and 87 proteins were present under both conditions.

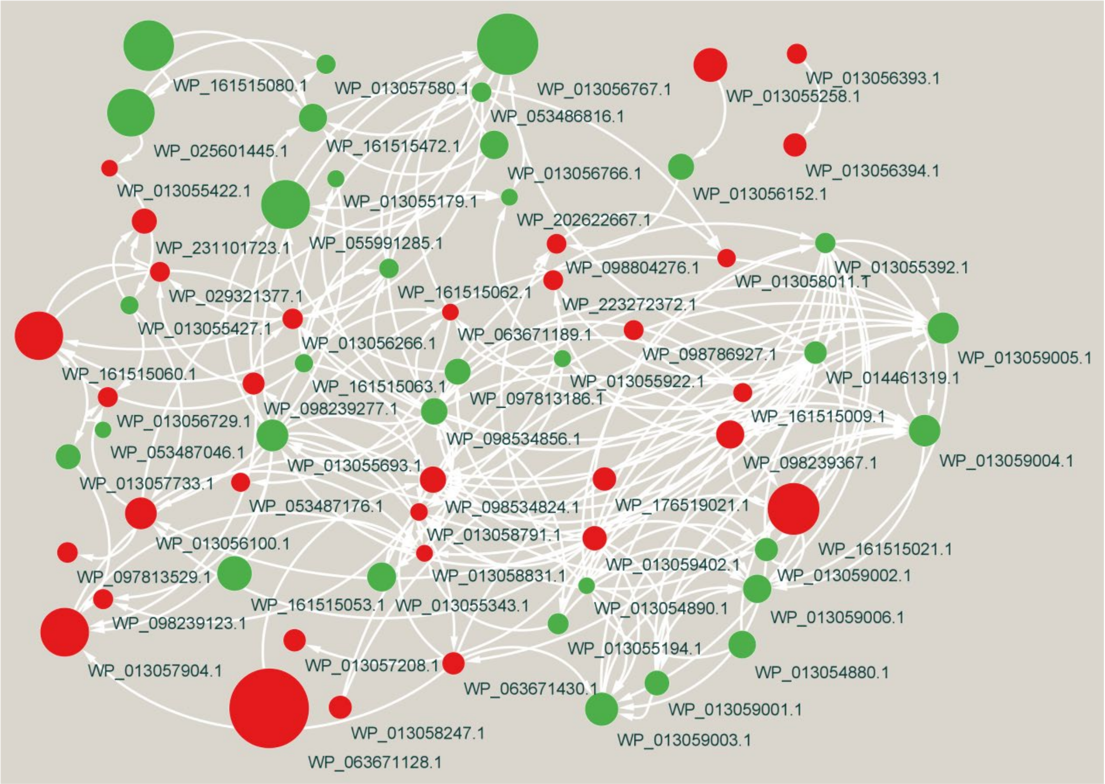

Figure S5. The protein and protein interaction networks of all differentially expressed proteins in N_2 treated sample. Up-(red) and down-(green) regulated DEPs are indicated. Size of the dot correlates to the absolute value of N_2 treated log2FC.

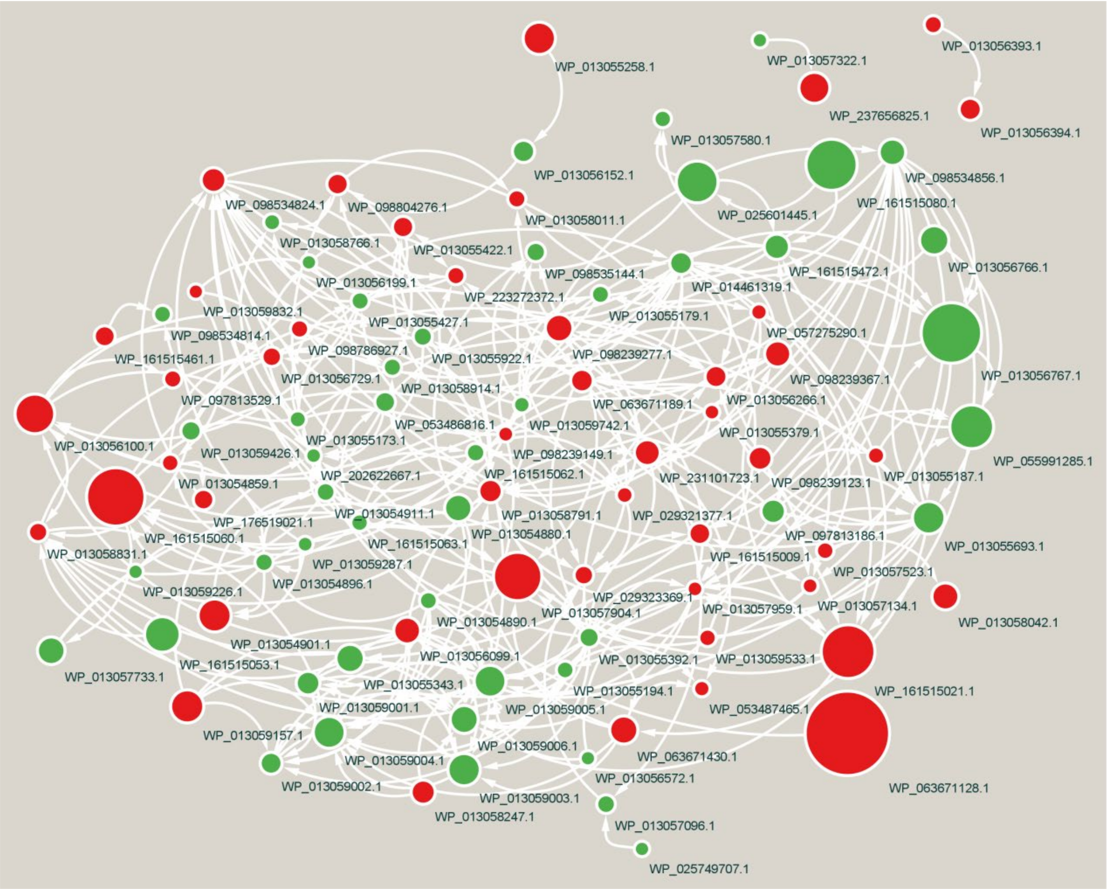

Figure S6. The protein and protein interaction networks of all differentially expressed proteins in the N_2-SF treated sample. Up-(red) and down-(green) regulated DEPs are shown. Size of the dot corelates to the absolute value of N_2-SF treated log2FC.

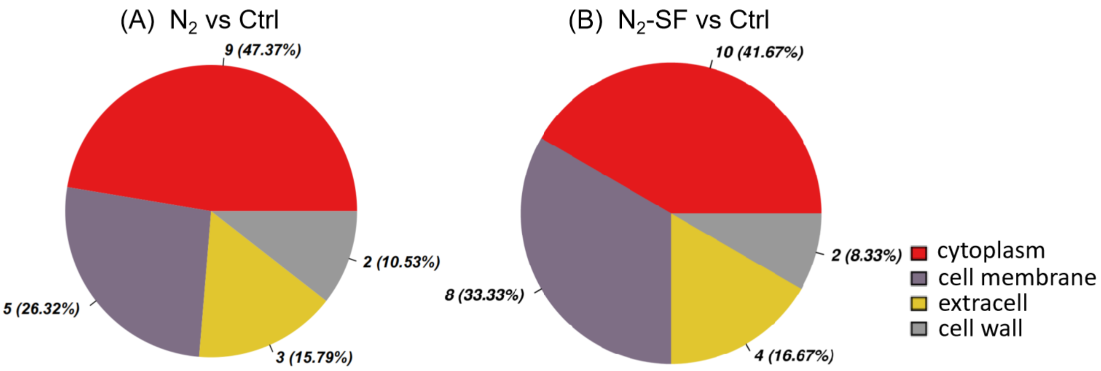

Figure S7. Subcellular location of differentially expressed proteins. N_2 and N_2-SF treated samples were analyzed and the proteins from different locations are shown in different color.

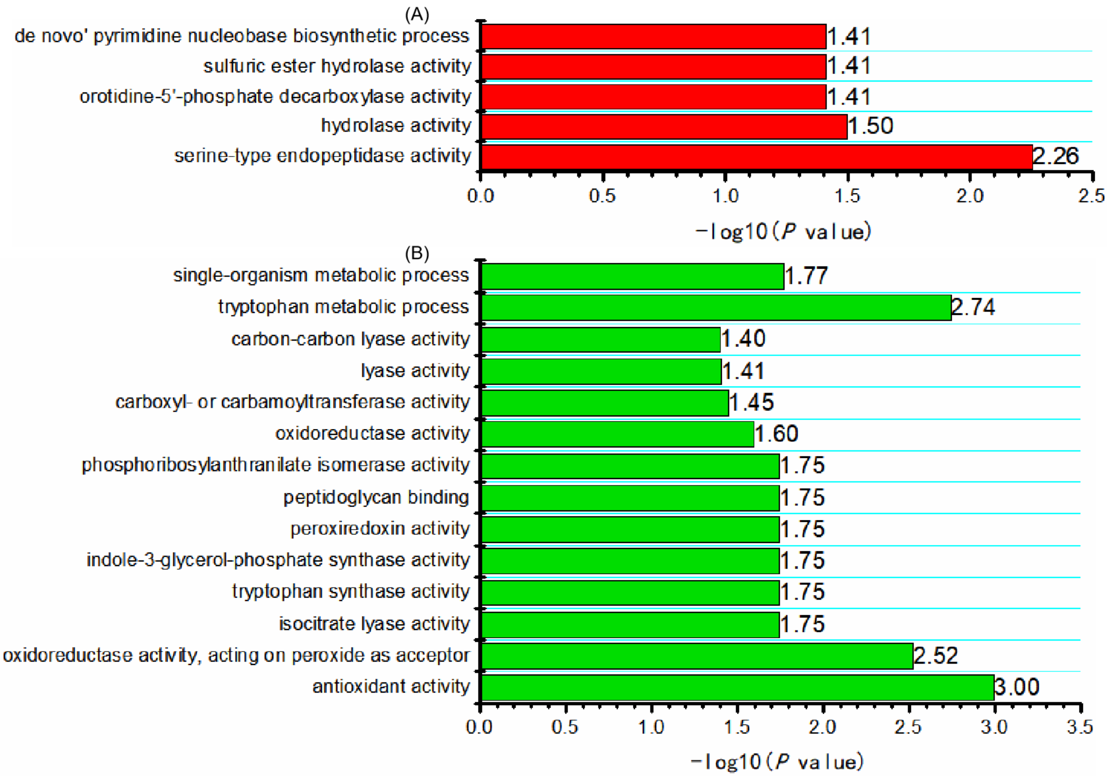

Figure S8 GO-based enrichment analysis of up-regulated proteins (A, red) and down-regulated proteins (B, green) in N_2 treated sample.

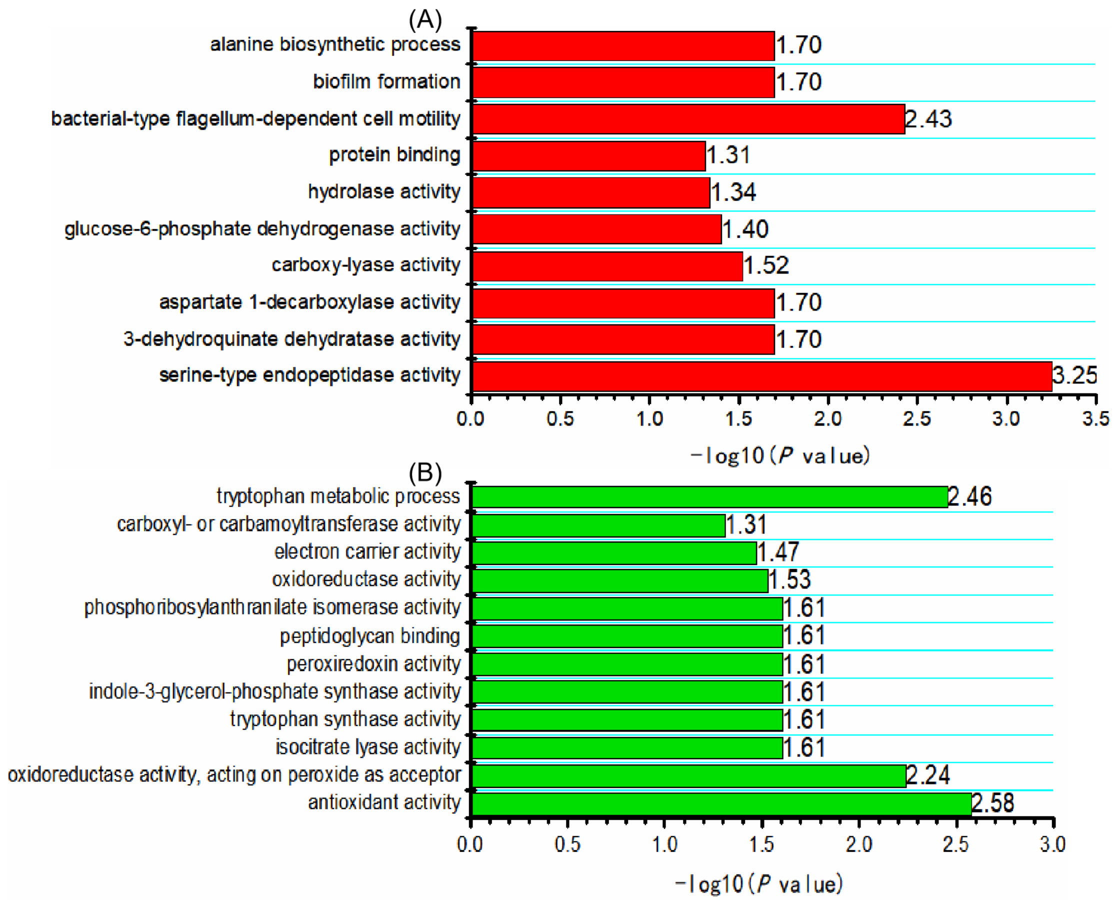

Figure S9 GO-based enrichment analysis of up-regulated (A, red) and down-regulated (B, green) proteins in the N_2-SF treated sample.

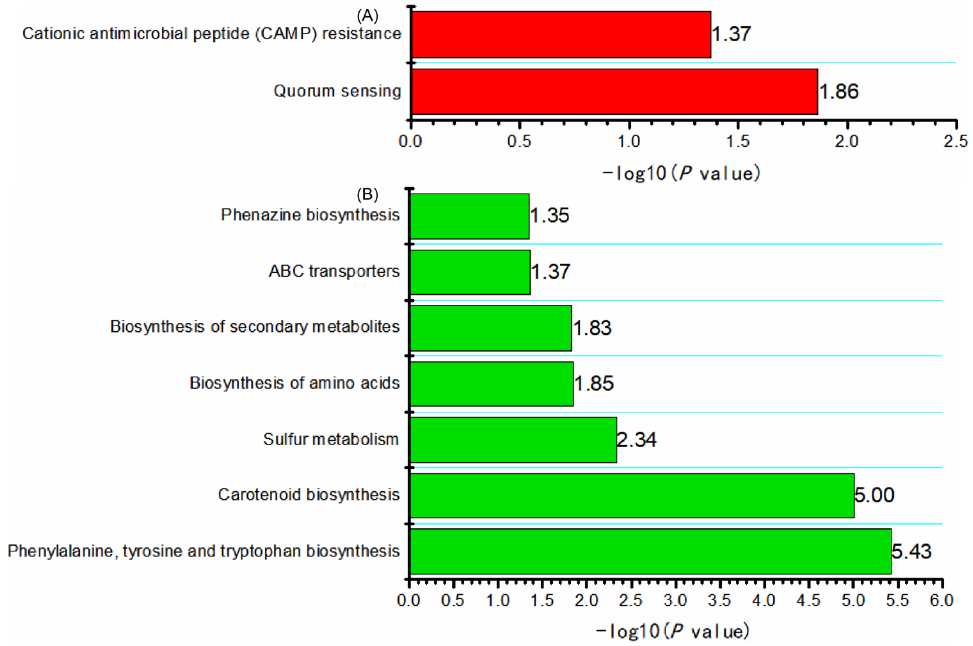

Figure S10 KEGG-based enrichment analysis of up-regulated (A, red) and down-regulated (B, green) proteins of N_2 treated sample.

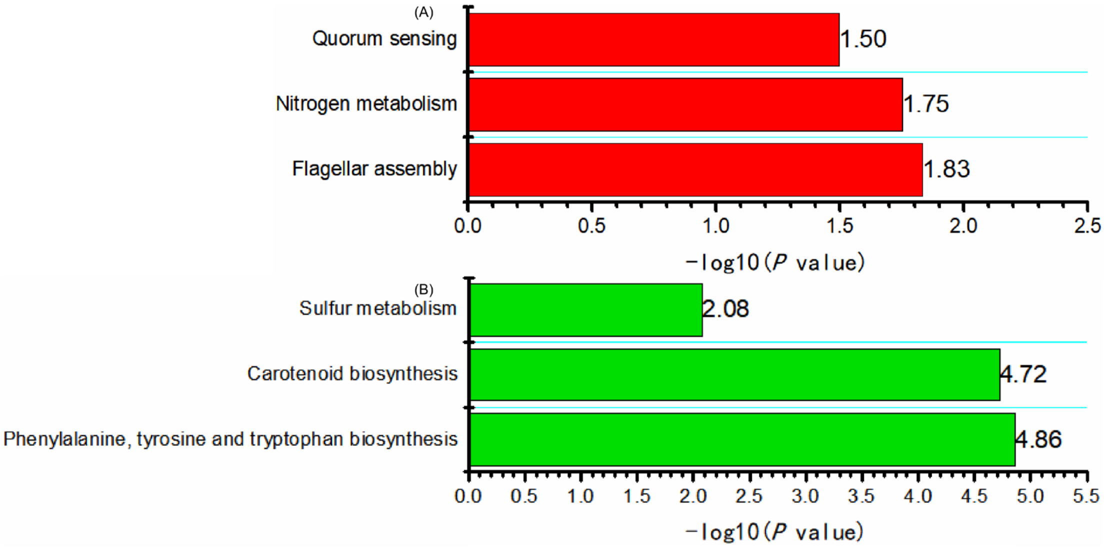

Figure S11 KEGG-based enrichment analysis of up-regulated (A, red) and down-regulated (B, green) proteins of N_2-SF treated sample.

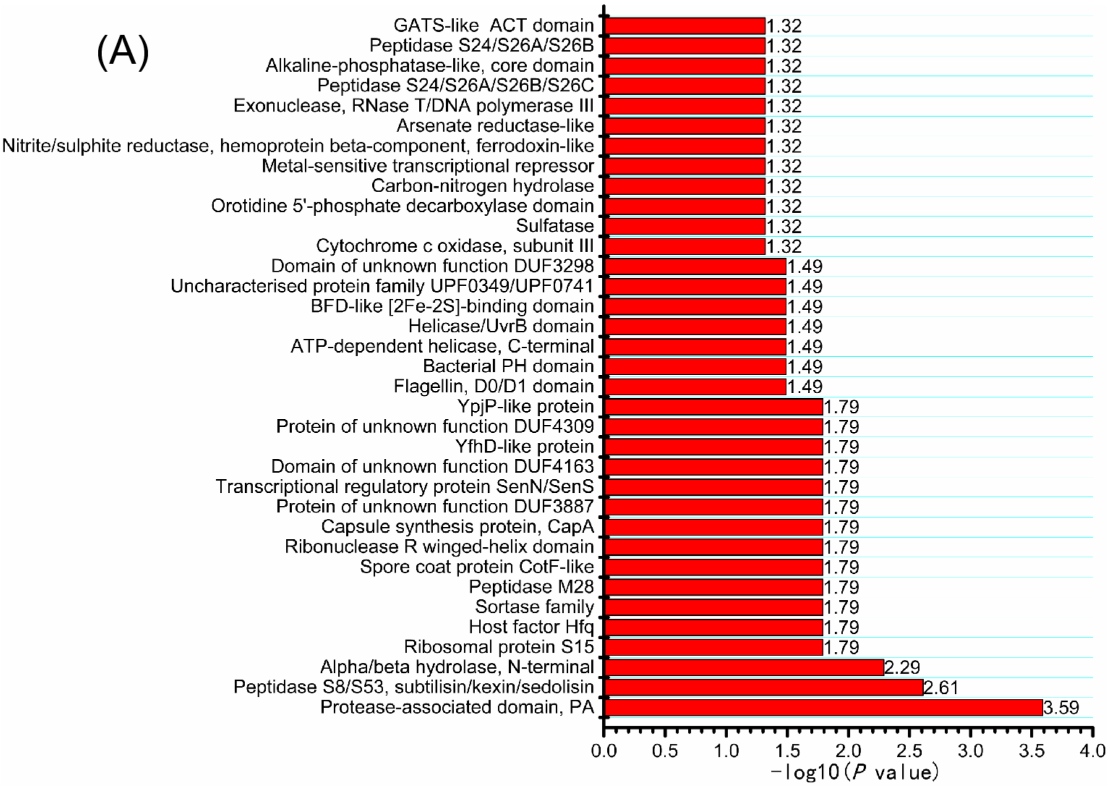

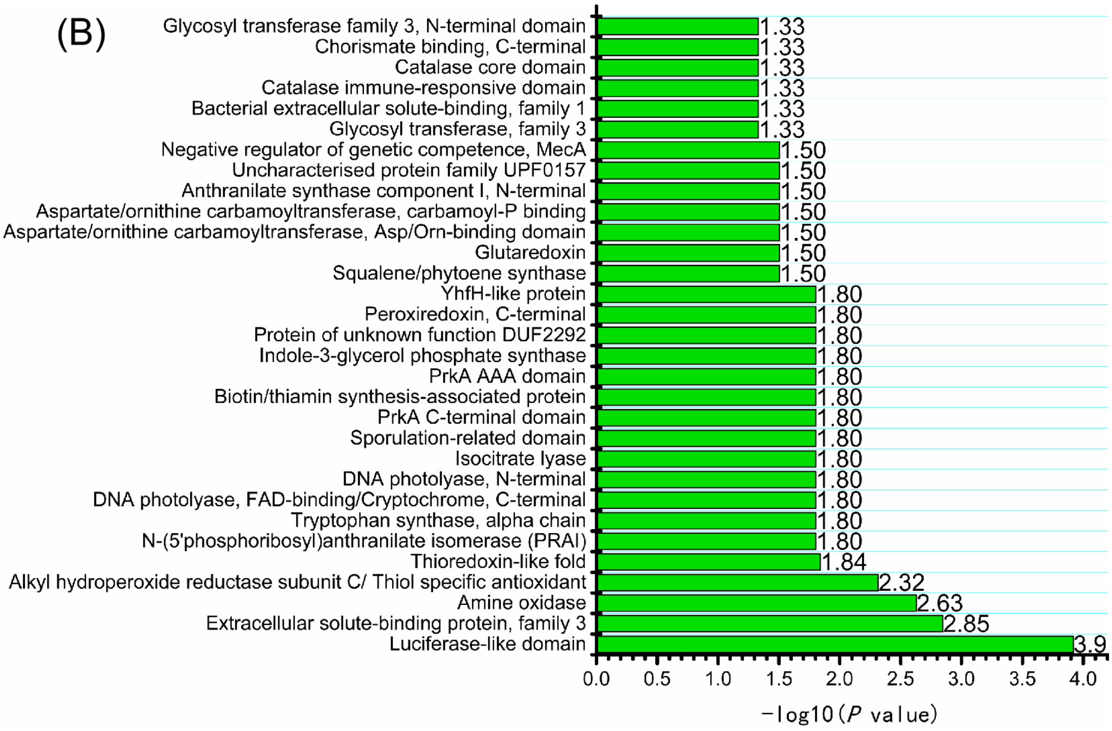

Figure S12 Protein domain-based enrichment analysis of up-regulated (A, red) and down-regulated (B, green) proteins of N_2 treated sample. The protein domains are indicated on the left side and the possibility values are indicated on the right side.

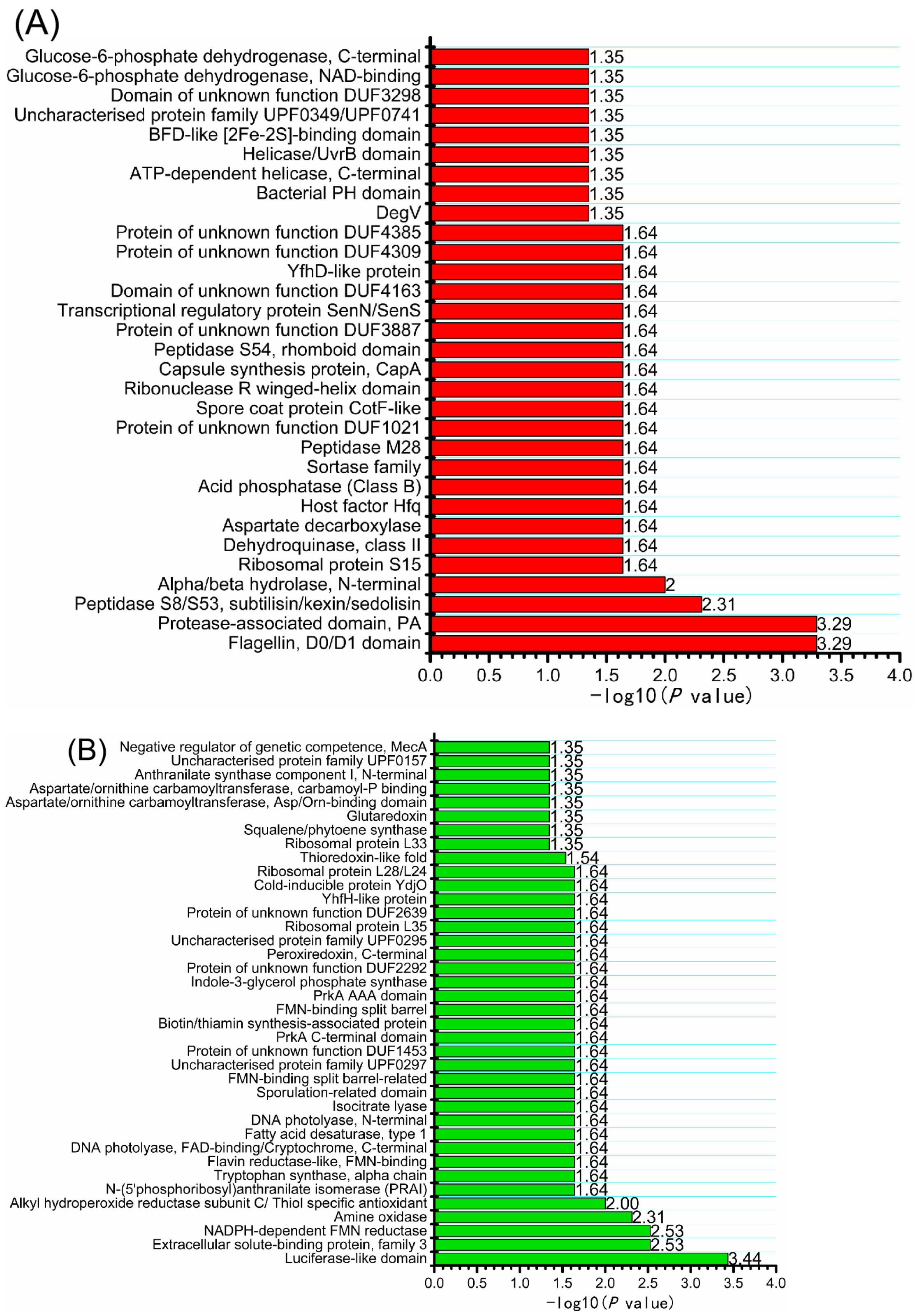

Figure S13 Protein domain-based enrichment analysis of up-regulated (A, red) and down-regulated (B, green) proteins of N_2-SF treated sample. The protein domains are indicated on the left side and the possibility values are indicated on the right side.

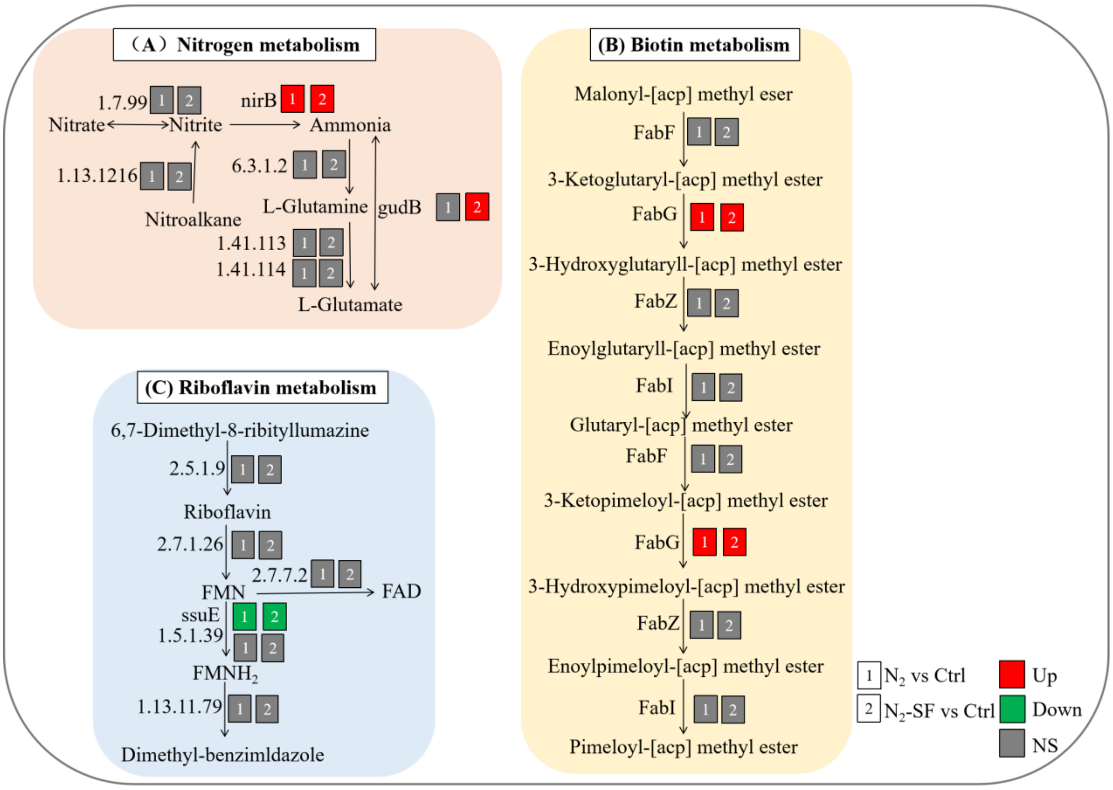

Figure S14 Schematic diagram of nitrogen metabolism, biotin metabolism and riboflavin metabolism. Colored boxes indicate the different expression profiles (upregulated, red; downregulated, green; no significance (NS), gray) under N_2 (1) and N_2-SF (2) conditions compared with cells without exposing to palladium. The information of upregulated or downregulated proteins involved in these cellular metabolisms is shown in the Supplementary Table S3.

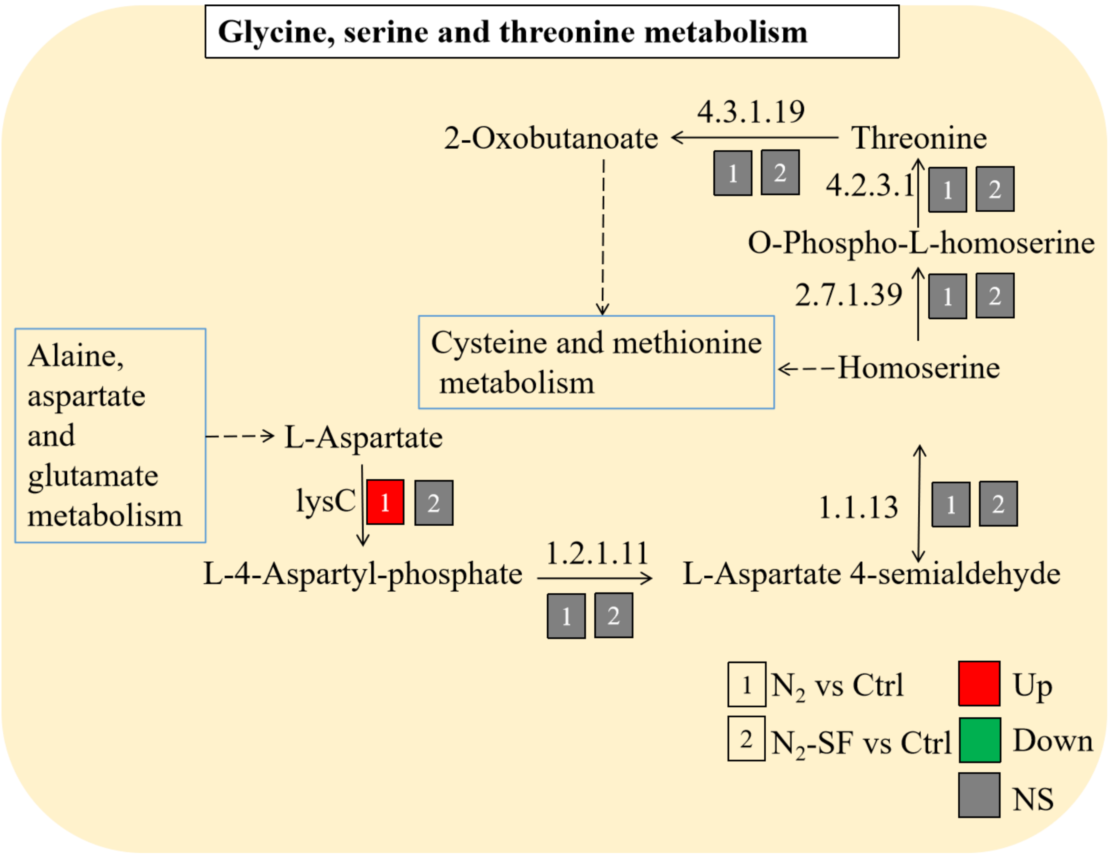

Figure S15 Schematic diagram of glycine, serine and threonine metabolism. Colored boxes indicate the different expression profiles. Upregulated, downregulated and no significantly (NS) changed pathways are shown in red, green and gray, respectively under N_2 (1) and N_2-SF (2) conditions compared with cells without exposing to palladium. The information of upregulated or downregulated proteins involved in these cellular metabolisms is shown in the Supplementary Table S4.

Table S3. The Pd (II) and Pd (0) XPS peak area of different samples.

	BE (eV)	N_2	N_2-SF
Pd (II)	342.3-343.4	1677.94	3230.23
	337.1-338.1	3404.1	3096.322
Pd (0)	340.0-340.7	351.31	1213.51
	334.7-335.4	879.56	4093.72

Table S4. All the identified quantifiable upregulated and downregulated proteins.

			N_2 vs Ctrl		N_2-SF vs Ctrl	
Protein ID	Protein	Description	FC	UP. DOWN	FC	UP. DOWN
WP_013054880.1	spoIIE	MULTISPECIES: stage II sporulation protein E [*Bacillaceae*]	0.70	down	0.68	down
WP_013054890.1	pabA	MULTISPECIES: aminodeoxychorismate/anthranilate synthase component II [*Bacillaceae*]	0.83	down	0.80	down
WP_013055132.1	-	MULTISPECIES: BH0509 family protein [*Bacillaceae]*	0.72	down	0.71	down
WP_013055179.1	-	MULTISPECIES: PrkA family serine protein kinase [*Bacillaceae*]	0.83	down	0.80	down
WP_013055194.1	bioB	MULTISPECIES: biotin synthase BioB [*Bacillaceae*]	0.78	down	0.80	down
WP_013055216.1	-	MULTISPECIES: hypothetical protein [*Bacillaceae*]	1.33	up	1.28	up
WP_013055258.1	-	MULTISPECIES: SurA N-terminal domain-containing protein [*Bacillaceae*]	1.58	up	1.60	up
WP_013055337.1	-	MULTISPECIES: YhfH family protein [*Bacillaceae*]	0.79	down	0.67	down
WP_013055343.1	aceA	MULTISPECIES: isocitrate lyase [*Bacillaceae*]	0.68	down	0.67	down
WP_013055392.1	argF	MULTISPECIES: ornithine carbamoyltransferase [*Bacillaceae*]	0.78	down	0.77	down
WP_013055422.1	spxA	MULTISPECIES: transcriptional regulator SpxA [*Bacillaceae*]	1.20	up	1.33	up
WP_013055427.1	mecA	MULTISPECIES: adaptor protein MecA [*Bacillaceae*]	0.81	down	0.79	down
WP_013055599.1	hxlA	MULTISPECIES: 3-hexulose-6-phosphate synthase [*Bacillaceae*]	1.42	up	1.43	up

WP_013055693.1	-	MULTISPECIES: TlpA family protein disulfide reductase [*Bacillaceae*]	0.65	down	0.62	down
WP_013055768.1	-	MULTISPECIES: YfhD family protein [*Bacillaceae*]	1.36	up	1.30	up
WP_013055922.1	-	MULTISPECIES: MerR family transcriptional regulator [*Bacillaceae*]	0.83	down	0.78	down
WP_013056022.1	-	MULTISPECIES: hypothetical protein [*Bacillaceae*]	1.24	up	NA	NA
WP_013056100.1	dinG	MULTISPECIES: ATP-dependent DNA helicase DinG [*Bacillaceae*]	1.53	up	1.81	up
WP_013056152.1	-	MULTISPECIES: extracellular solute-binding protein [*Bacillaceae*]	0.72	down	0.74	down
WP_013056266.1	-	MULTISPECIES: L-threonine 3-dehydrogenase [*Bacillaceae*]	1.28	up	1.34	up
WP_013056393.1	-	MULTISPECIES: hypothetical protein [*Bacillaceae*]	1.27	up	1.26	up
WP_013056394.1	-	MULTISPECIES: spore coat protein [*Bacillaceae*]	1.34	up	1.35	up
WP_013056729.1	-	MULTISPECIES: LTA synthase family protein [*Bacillaceae*]	1.27	up	1.29	up
WP_013056766.1	-	MULTISPECIES: GNAT family N-acetyltransferase [*Bacillaceae*]	0.69	down	0.66	down
WP_013056767.1	-	MULTISPECIES: glutaredoxin family protein [*Bacillaceae*]	0.41	down	0.39	down
WP_013056824.1	-	MULTISPECIES: YezD family protein [*Bacillaceae*]	0.63	down	0.57	down
WP_013057208.1	-	MULTISPECIES: alpha/beta hydrolase [*Bacillaceae*]	1.32	up	1.46	up
WP_013057252.1	-	MULTISPECIES: FbpB family small basic protein [*Bacillaceae*]	1.88	up	1.99	up
WP_013057277.1	-	MULTISPECIES: hypothetical protein [*Bacillaceae*]	0.62	down	0.59	down
WP_013057464.1	-	MULTISPECIES: hypothetical protein [*Bacillaceae*]	1.31	up	1.32	up
WP_013057500.1	-	MULTISPECIES: CapA family protein [*Bacillaceae*]	1.27	up	1.31	up

WP_013057580.1	ssuD	MULTISPECIES: FMNH2-dependent alkanesulfonate monooxygenase [*Bacillaceae*]	0.80	down	0.80	down
WP_013057733.1	-	MULTISPECIES: GrpB family protein [*Bacillaceae*]	0.73	down	0.68	down
WP_013057904.1	lepB	MULTISPECIES: signal peptidase I [*Bacillaceae*]	2.00	up	2.11	up
WP_013057999.1	-	MULTISPECIES: gas vesicle protein GvpP [*Bacillaceae*]	1.25	up	1.22	up
WP_013058011.1	-	MULTISPECIES: alpha/beta fold hydrolase [*Bacillaceae*]	1.24	up	1.26	up
WP_013058042.1	-	MULTISPECIES: metal-sensitive transcriptional regulator [*Bacillaceae*]	1.25	up	1.46	up
WP_013058247.1	-	MULTISPECIES: DUF3298 and DUF4163 domain-containing protein [*Bacillaceae*]	1.33	up	1.39	up
WP_013058550.1	-	MULTISPECIES: hypothetical protein [*Bacillaceae*]	1.44	up	1.43	up
WP_013058791.1	hfq	MULTISPECIES: RNA chaperone Hfq [*Bacillaceae*]	1.22	up	1.38	up
WP_013058831.1	rpsO	MULTISPECIES: 30S ribosomal protein S15 [*Bacillaceae*]	1.21	up	1.28	up
WP_013059001.1	trpA	MULTISPECIES: tryptophan synthase subunit alpha [*Bacillaceae*]	0.73	down	0.72	down
WP_013059002.1	trpB	MULTISPECIES: tryptophan synthase subunit beta [*Bacillaceae*]	0.75	down	0.75	down
WP_013059003.1	-	MULTISPECIES: phosphoribosylanthranilate isomerase [*Bacillaceae*]	0.64	down	0.62	down
WP_013059004.1	trpC	MULTISPECIES: indole-3-glycerol phosphate synthase TrpC [*Bacillaceae*]	0.66	down	0.63	down
WP_013059005.1	trpD	MULTISPECIES: anthranilate phosphoribosyltransferase [*Bacillaceae*]	0.66	down	0.63	down
WP_013059006.1	trpE	MULTISPECIES: anthranilate synthase component I [*Bacillaceae*]	0.69	down	0.67	down

WP_013059402.1	-	MULTISPECIES: aspartate kinase [*Bacillaceae*]	1.36	up	NA	NA
WP_013059850.1	-	MULTISPECIES: DUF1450 domain-containing protein [*Bacillaceae*]	1.29	up	1.21	up
WP_014461319.1	-	MULTISPECIES: peroxiredoxin [*Bacillaceae*]	0.76	down	0.74	down
WP_025601445.1	ssuE	MULTISPECIES: NADPH-dependent FMN reductase [*Bacillaceae*]	0.51	down	0.53	down
WP_025752035.1	-	MULTISPECIES: YpjP family protein [*Bacillaceae*]	1.22	up	NA	NA
WP_025752355.1	-	MULTISPECIES: hypothetical protein [*Bacillaceae*]	0.81	down	0.79	down
WP_028413131.1	-	MULTISPECIES: hypothetical protein [*Bacillaceae*]	0.83	down	0.80	down
WP_029321377.1	-	MULTISPECIES: M28 family peptidase [*Bacillaceae*]	1.27	up	1.23	up
WP_029325566.1	-	MULTISPECIES: hypothetical protein [*Bacillaceae*]	0.80	down	0.74	down
WP_053486816.1	-	MULTISPECIES: phytoene/squalene synthase family protein [*Bacillaceae*]	0.79	down	0.76	down
WP_053487046.1	-	MULTISPECIES: iron-hydroxamate ABC transporter substrate-binding protein [*Bacillaceae*]	0.83	down	NA	NA
WP_053487162.1	-	MULTISPECIES: hypothetical protein [*Bacillaceae*]	0.75	down	0.69	down
WP_053487176.1	cyoC	MULTISPECIES: cytochrome o ubiquinol oxidase subunit III [*Bacillaceae*]	1.25	up	NA	NA
WP_053487753.1	-	MULTISPECIES: SPOR domain-containing protein [*Bacillaceae*]	0.82	down	0.81	down
WP_055991285.1	-	MULTISPECIES: amino acid ABC transporter substrate-binding protein [*Bacillaceae*]	0.50	down	0.51	down
WP_061859275.1	-	MULTISPECIES: hypothetical protein [*Bacillaceae*]	0.81	down	0.79	down

WP_063671128.1	-	MULTISPECIES: sigma 54-interacting transcriptional regulator [*Bacillaceae*]	3.26	up	3.88	up
WP_063671189.1	-	MULTISPECIES: LysR family transcriptional regulator [*Bacillaceae*]	1.20	up	1.36	up
WP_063671430.1	-	MULTISPECIES: allantoinase [*Bacillaceae*]	1.32	up	1.49	up
WP_078082119.1	-	MULTISPECIES: hypothetical protein [*Bacillaceae*]	1.66	up	1.45	up
WP_097813186.1	-	MULTISPECIES: transporter substrate-binding domain-containing protein [*Bacillaceae*]	0.72	down	0.71	down
WP_097813529.1	-	MULTISPECIES: C40 family peptidase [*Bacillaceae*]	1.28	up	1.26	up
WP_098239123.1	-	MULTISPECIES: SDR family oxidoreductase [Bacillaceae]	1.27	up	1.39	up
WP_098239277.1	-	carbon-nitrogen hydrolase family protein [*Priestia megaterium*]	1.31	up	1.48	up
WP_098239367.1	-	MULTISPECIES: S8 family peptidase [*Bacillaceae*]	1.44	up	1.44	up
WP_098534824.1	-	trypsin-like peptidase domain-containing protein [*Priestia megaterium*]	1.40	up	1.41	up
WP_098534856.1	katA	MULTISPECIES: catalase KatA [*Bacillaceae*]	0.71	down	0.69	down
WP_098786927.1	nirB	nitrite reductase large subunit NirB [*Priestia megaterium*]	1.27	up	1.26	up
WP_098804276.1	-	class D sortase [*Priestia megaterium*]	1.26	up	1.33	up
WP_161515009.1	galU	UTP--glucose-1-phosphate uridylyltransferase GalU [*Priestia megaterium*]	1.25	up	1.34	up
WP_161515021.1	-	flagellin [Priestia megaterium]	2.10	up	2.30	up
WP_161515053.1	-	homocysteine synthase [*Priestia megaterium*]	0.63	down	0.59	down
WP_161515060.1	-	M20/M25/M40 family metallo-hydrolase [*Priestia megaterium*]	1.99	up	2.49	up

WP_161515062.1	crtI	phytoene desaturase family protein [*Priestia megaterium*]	0.79	down	0.79	down
WP_161515063.1	crtI	phytoene desaturase family protein [*Priestia megaterium*]	0.81	down	0.80	down
WP_161515080.1	ssuD	FMNH2-dependent alkanesulfonate monooxygenase [*Priestia megaterium*]	0.48	down	0.45	down
WP_161515461.1	esaA	type VII secretion protein EsaA [*Priestia megaterium*]	1.25	up	1.32	up
WP_161515472.1	-	LLM class flavin-dependent oxidoreductase [*Priestia megaterium*]	0.69	down	0.70	down
WP_161515536.1	-	LPXTG cell wall anchor domain-containing protein [*Priestia megaterium*]	1.27	up	1.31	up
WP_176519021.1	-	3D domain-containing protein, partial [*Priestia megaterium*]	1.34	up	1.31	up
WP_194719226.1	-	YjgB family protein [*Priestia megaterium*]	1.40	up	1.46	up
WP_202622667.1	-	DNA photolyase family protein [*Priestia megaterium*]	0.83	down	0.83	down
WP_223272372.1	-	MULTISPECIES: S8 family serine peptidase [*Bacillaceae*]	1.27	up	1.26	up
WP_231101723.1	-	PH domain-containing protein [*Priestia megaterium*]	1.38	up	1.43	up
WP_237656825.1	-	hypothetical protein [*Priestia megaterium*]	1.45	up	1.57	up
WP_013054859.1	-	MULTISPECIES: Veg family protein [*Bacillaceae*]	NA	NA	1.25	up
WP_013054896.1	-	MULTISPECIES: UvrB/UvrC motif-containing protein [*Bacillaceae*]	NA	NA	0.79	down
WP_013054901.1	-	MULTISPECIES: PIN/TRAM domain-containing protein [*Bacillaceae*]	NA	NA	1.62	up
WP_013054911.1	rpmG	MULTISPECIES: 50S ribosomal protein L33 [*Bacillaceae*]	NA	NA	0.79	down
WP_013055173.1	-	MULTISPECIES: YgzB family protein [*Bacillaceae*]	NA	NA	0.81	down

WP_013055187.1	-	MULTISPECIES: cation:dicarboxylase symporter family transporter [*Bacillaceae*]	NA	NA	1.24	up
WP_013055224.1	iolE	MULTISPECIES: myo-inosose-2 dehydratase [*Bacillaceae*]	NA	NA	1.22	up
WP_013055379.1	-	MULTISPECIES: DegV family protein [*Bacillaceae*]	NA	NA	1.21	up
WP_013055551.1	-	MULTISPECIES: helix-turn-helix transcriptional regulator [*Bacillaceae*]	NA	NA	0.81	down
WP_013055752.1	-	MULTISPECIES: hypothetical protein [*Bacillaceae*]	NA	NA	1.20	up
WP_013055966.1	-	MULTISPECIES: aspartyl-phosphate phosphatase Spo0E family protein [*Bacillaceae*]	NA	NA	1.25	up
WP_013056099.1	panD	MULTISPECIES: aspartate 1-decarboxylase [*Bacillaceae*]	NA	NA	1.46	up
WP_013056199.1	-	MULTISPECIES: fatty acid desaturase [*Bacillaceae*]	NA	NA	0.83	down
WP_013056572.1	qoxB	MULTISPECIES: cytochrome aa3 quinol oxidase subunit I [*Bacillaceae*]	NA	NA	0.83	down
WP_013056990.1	-	MULTISPECIES: cold-shock protein [*Bacillaceae*]	NA	NA	0.82	down
WP_013057019.1	-	MULTISPECIES: hypothetical protein [*Bacillaceae*]	NA	NA	0.83	down
WP_013057096.1	-	MULTISPECIES: MarR family transcriptional regulator [*Bacillaceae*]	NA	NA	0.79	down
WP_013057134.1	-	MULTISPECIES: Glu/Leu/Phe/Val dehydrogenase [*Bacillaceae*]	NA	NA	1.21	up
WP_013057322.1	-	MULTISPECIES: hypothetical protein [*Bacillaceae*]	NA	NA	0.83	down
WP_013057523.1	-	MULTISPECIES: DUF4385 domain-containing protein [*Bacillaceae*]	NA	NA	1.25	up
WP_013057959.1	zwf	MULTISPECIES: glucose-6-phosphate dehydrogenase [*Bacillaceae*]	NA	NA	1.21	up

WP_013058698.1	-	MULTISPECIES: ATP-binding cassette domain-containing protein [*Bacillaceae*]	NA	NA	1.24	up
WP_013058766.1	-	MULTISPECIES: cytochrome c biogenesis protein CcdC [*Bacillaceae*]	NA	NA	0.81	down
WP_013058914.1	rpmB	MULTISPECIES: 50S ribosomal protein L28 [*Bacillaceae*]	NA	NA	0.79	down
WP_013059157.1	aroQ	MULTISPECIES: type II 3-dehydroquinate dehydratase [*Bacillaceae*]	NA	NA	1.62	up
WP_013059226.1	-	MULTISPECIES: cytidine deaminase [*Bacillaceae*]	NA	NA	0.83	down
WP_013059287.1	-	MULTISPECIES: IreB family regulatory phosphoprotein [*Bacillaceae*]	NA	NA	0.83	down
WP_013059426.1	rpmI	MULTISPECIES: 50S ribosomal protein L35 [*Bacillaceae*]	NA	NA	0.77	down
WP_013059533.1	-	MULTISPECIES: gamma carbonic anhydrase family protein [*Bacillaceae*]	NA	NA	1.25	up
WP_013059742.1	-	MULTISPECIES: flavodoxin family protein [*Bacillaceae*]	NA	NA	0.82	down
WP_013059832.1	-	MULTISPECIES: TetR/AcrR family transcriptional regulator [*Bacillaceae*]	NA	NA	1.20	up
WP_016762840.1	-	MULTISPECIES: YflJ family protein [*Bacillaceae*]	NA	NA	0.71	down
WP_025749707.1	-	MULTISPECIES: flavin reductase family protein [*Bacillaceae*]	NA	NA	0.83	down
WP_029323369.1	kynU	MULTISPECIES: kynureninase [*Bacillaceae*]	NA	NA	1.29	up
WP_053487465.1	-	MULTISPECIES: 5-nucleotidase, lipoprotein e(P4) family [*Bacillaceae*]	NA	NA	1.22	up
WP_053487569.1	-	MULTISPECIES: hypothetical protein [*Bacillaceae*]	NA	NA	0.76	down
WP_057275290.1	flgL	MULTISPECIES: flagellar hook-associated protein FlgL [*Bacillaceae*]	NA	NA	1.22	up

WP_063671674.1	-	MULTISPECIES: hypothetical protein [*Bacillaceae*]	NA	NA	0.82	down
WP_098239149.1	-	rhomboid family intramembrane serine protease [*Priestia megaterium*]	NA	NA	1.21	up
WP_098534814.1	-	putative metal-dependent hydrolase [*Priestia megaterium*]	NA	NA	0.80	down
WP_098535144.1	-	LysR family transcriptional regulator [*Priestia megaterium*]	NA	NA	0.78	down
WP_013058976.1	rpmF	MULTISPECIES: 50S ribosomal protein L32 [*Bacillaceae*]	NA	NA	NA	NA

References

1. Marx, H.; Minogue, C.E.; Jayaraman, D.; Richards, A.L.; Kwiecien, N.W.; Siahpirani, A.F.; Rajasekar, S.; Maeda, J.; Garcia, K.; Del Valle-Echevarria, A.R.; Volkening, J.D.; Westphall, M.S.; Roy, S.; Sussman, M.R.; Ane, J.; Coon, J.J. A proteomic atlas of the legume Medicago truncatula and its nitrogen-fixing endosymbiont Sinorhizobium meliloti. *Nat. Biotechnol.* **2016**, *34*, 1198.
2. Nie, L.; Zhang, H.; Wu, Z.; Wang, Y.; Liu, H.; Wu, X.; Wang, W. Modified TCA/acetone precipitation of plant proteins for proteomic analysis. *PLoS ONE* **2018**, *13*.
3. Bradford, M.M. A rapid and sensitive method for the quantitation of microgram quantities of protein utilizing the principle of protein-dye binding. *Anal. Biochem.* **1976**, *72*, 248-254.
4. Kachuk, C.; Stephen, K.; Doucette, A. Comparison of sodium dodecyl sulfate depletion techniques for proteome analysis by mass spectrometry. *J. Chromatogr. A* **2015**, *1418*, 158-166.
5. Wisniewski, J.R.; Zougman, A.; Nagaraj, N.; Mann, M.Universal sample preparation method for proteome analysis. *Nat. Methods* **2009**, *6*, 359-360.
6. Gillette, M.A.; Satpathy, S.; Cao, S.; Dhanasekaran, S.M.; Vasaikar, S.V.; Krug, K.; Petralia, F.; Li, Y.Z.; Liang, W.W.; Reva, B.; Krek, A.; Ji, J.Y.; Song, X.Y.; Liu, W.K.; Hong, R.Y.; Yao, L.J.; Blumenberg, L.; Savage, S.R.; Wendl, M.C.; Wen, B.; Li, K.; Tang, L.C.; MacMullan, M.A.; Avanessian, S.C.; Kane, M.H.; Newton, C.J.; Cornwell, M.; Kothadia, R.B.; Ma, W.P.; Yoo, S.; Mannan, R.; Vats, P.; Kumar-Sinha, C.; Kawaler, E.A.; Omelchenko, T.; Colaprico, A.; Geffen, Y.; Maruvka, Y.E.; Leprevost, F.D.; Wiznerowicz, M.; Gumus, Z.H.; Veluswamy, R.R.; Hostetter, G.; Heiman, D.I.; Wyczalkowski, M.A.; Hiltke, T.; Mesri, M.; Kinsinger, C.R.; Boja, E.S.; Omenn, G.S.; Chinnaiyan, A.M.; Rodriguez, H.; Li, Q.K.; Jewell, S.D.; Thiagarajan, M.; Getz, G.; Zhang, B.; Fenyo, D.; Ruggles, K.V.; Cieslik, M.P.; Robles, A.I.; Clauser, K.R.; Govindan, R.; Wang, P.; Nesvizhskii, A.I.; Ding, L.; Mani, D.R.; Carr, S.A., Clinical PTAC. Proteogenomic Characterization Reveals Therapeutic Vulnerabilities in Lung Adenocarcinoma. *Cell* **2020**, *182*, 200.
